# Metabolome-wide associations with short-term exposure to PM_2.5_-bound polycyclic aromatic hydrocarbons: a study in older adults

**DOI:** 10.3389/fpubh.2025.1609724

**Published:** 2025-07-24

**Authors:** Haoneng Hu, Quan Zhou, Kang Cao, Yu Jiang, Jianjun Xiang, Jing Wu, Jin Li, Zhiwei Chen, Shuling Kang, Dandan Zhu, Huaying Lin, Chuancheng Wu

**Affiliations:** ^1^Department of Preventive Medicine, School of Public Health, Fujian Medical University, Fuzhou, China; ^2^Fujian Medical University Affiliated Center for Disease Control and Prevention, Fuzhou, China; ^3^Quanzhou Medical College, Quanzhou, China

**Keywords:** PM_2.5_, PAHs, metabolomics, metabolic pathway, older adults

## Abstract

**Background:**

Emerging evidence links fine particulate matter (PM_2.5_) and its polycyclic aromatic hydrocarbon (PAH) components to adverse health outcomes. However, the biological mechanisms driving these associations remain unclear. This study innovatively integrates personal exposure monitoring and untargeted metabolomics in an older adult population to investigate the differential impacts of individual PM_2.5_-bound PAHs on metabolic pathways and elucidate their roles in health risks.

**Methods:**

In this study, we enlisted the participation of 112 healthy older adults. We employed personal samplers to monitor the concentrations of pollutants throughout the study period. Furthermore, we conducted an untargeted metabolomic analysis of plasma samples using a liquid chromatograph mass spectrometer (LC–MS). A general linear regression model was utilized to investigate the significant relationships between metabolites and pollutants. Metabolic pathway enrichment analysis was performed to reveal the disturbed metabolic pathways related to PM_2.5_-bound PAHs.

**Results:**

Our study demonstrated that short-term exposure to PM_2.5_-bound PAHs may induce acute perturbations in plasma metabolites among the older adult population. We found that exposure to LMW PAHs in PM_2.5_ were correlated with amino acid metabolic pathways, while HMW-PAHs are associated with fatty acid and cholesterol metabolism pathways. While PM_2.5_ mass was higher in summer, the toxic PAHs component of PM_2.5_ was substantially higher in winter, contributing to greater observed toxicity.

**Conclusion:**

The plasma metabolome presents a promising resource for biomarkers and pathways, elucidating the biological mechanisms of PM_2.5_-bound PAHs. Our findings suggest that the cholesterol and citric acid metabolites, as well as the cholesterol biosynthesis and citric acid cycle pathways they affect, may play important roles in the health damage caused by PAHs, providing potential insights into the pathogenic processes underlying the impact of PM_2.5_-bound PAHs.

## 1 Introduction

Air pollution is widely acknowledged as a significant global environmental and public health threat, with particulate matter playing a pivotal role in the deterioration of air quality (1, 2). The WHO estimated that 4.2 million deaths annually could be attributed to PM_2.5_, with half of the world’s population exposed to increasing air pollution (3). The harmful effects of PM_2.5_ are attributed to its complex chemical composition (4). A multicenter longitudinal study indicated that even exposure to PM_2.5_ below established limits is associated with non-accidental mortality. The most significant effects were observed at lower concentrations, suggesting a concentration-response curve without an identifiable threshold (5). Another study demonstrated consistent increases in daily mortality with increasing PM concentration, with steeper slopes observed at lower PM concentrations (6).

Polycyclic aromatic hydrocarbons (PAHs) adsorbed on PM_2.5_, characterized by their multiple benzene rings, are persistent organic pollutants and primary atmospheric contaminants. They are known for their carcinogenic, teratogenic, and endocrine-disrupting properties, and were among the initial atmospheric pollutants identified as suspected carcinogens (7). The distribution, transformation, and bioaccumulation effects of these compounds in the atmospheric environment represent a significant threat to the environment and human health (8, 9). Several studies have linked exposure to PM_2.5_-borne PAHs to altered lipid profiles (10, 11), inflammatory reactions, and oxidative stress (12–15). Some research also suggests a correlation between exposure to these compounds and adverse health effects such as lung cancer, cardiovascular disease, respiratory non-malignant diseases like asthma and chronic obstructive pulmonary disease, diabetes, and obesity (16–19). PAHs typically exist as a group, with different components exhibiting varying levels of toxicity. Based on their molecular weight, they can be classified into low molecular weight PAHs and high molecular weight PAHs. Studies have demonstrated that as the molecular weight increases, the carcinogenicity of PAHs rises while acute toxicity declines. Consequently, understanding the distinctive characteristics that may exist within PAH mixtures can facilitate the accurate assessment of their hazards and the implementation of risk control measures (20).

Despite numerous studies investigating the adverse health effects of PM_2.5_ and its chemical constituents, further understanding is required regarding the potential health risks of PM_2.5_ concentrations below established threshold limits, as well as the detrimental health effects of its individual components (4). Additionally, the mechanisms underlying these effects and the extent to which PAHs contribute to PM_2.5_’s health impacts remain unclear. The composition and concentration of particulate matter are fundamental characteristics that determine its toxicity and health effects. The exposure-effect relationship resulting from particulate matter exposure is complex, making it crucial to study the health impacts of its various components.

Air pollution epidemiology studies frequently use outdoor concentrations of ambient fine particulate matter as exposure surrogates, which can lead to exposure misclassification errors (21). This approach may overlook several factors, resulting in inaccurate pollutant exposure assessments. For example, it fails to account for individual exposure to air pollutants in various indoor and outdoor environments and daily activity patterns. Numerous studies have emphasized the importance of personal monitoring for a more comprehensive assessment of exposure and health risks, as personal monitoring can provide more precise measurements of individual exposure, especially for subjects with similar economic, environmental, and behavioral characteristics (22–24).

Metabolites in body fluids, which often reflect the ultimate outcomes of biological processes, offer insights into functional changes induced by external exposures (25–27). Metabolomics is a valuable platform that provides a global profiling of complex metabolic alterations in biological fluids or tissue samples after various perturbations, such as pathological states or exposure to external stimuli (28, 29). While air pollution affects individuals across all regions, ages, and social classes, certain subgroups—such as the older adult, children, and patients with cardiovascular or respiratory diseases—are considered more susceptible to its adverse health effects (30). There is substantial evidence indicating that individuals aged 65 and older are more vulnerable to the health impacts of air pollution compared to younger people, due to diminished physiological, metabolic, and compensatory processes, which increase their sensitivity to pollutants and elevate the incidence of related diseases (31). As the global population continues to age, addressing how to protect the older adult from pollution exposure and promote healthier living has become a matter of urgent international concern.

In this study, we employed an untargeted metabolomics approach to measure plasma metabolites in the older adult population and estimated exposure more accurately based on individual monitoring. Our objectives were to comprehensively investigate the relationships between personal PM_2.5_-bound PAH exposure and plasma metabolites in older adults, and to identify crucial biomarkers and potential metabolic pathways. This may shed light on the mechanisms by which PM_2.5_-bound PAHs lead to adverse health effects.

## 2 Materials and methods

### 2.1 Study participants and sample collection

This study is based on the ‘Air Pollution (Haze) Impact on Population Health and Surveillance and Protection’ project conducted by the Fuzhou Center for Disease Control and Prevention. Fuzhou serves as a clean air control site representing the exposure conditions of relatively low PM_2.5_ pollution areas. Study volunteers were enrolled from a community located at the urban–rural intersection in Fuzhou, located approximately 2 km away from the national air monitoring station, with no nearby industrial emission sources. We established an observational study in December 2020 and conducted follow-ups every summer (July–September) and winter (December–February). The study participants met the following criteria (1): healthy senior individuals aged ≥60 years residing in the community (2), having lived at the current residential address for more than 3 years and having no travel plans during the study period (3), voluntary participation and ability to complete the questionnaire (4), absence of chronic metabolic diseases or major diseases (e.g., diabetes mellitus, respiratory disease, cardiovascular or cerebrovascular diseases, hyperglycemia, hyperlipidemia, hypertension, cancers, or tumors), and (5) no occupational exposure to PAHs. For the present study, we only analyzed data from the period of 2020–2021.

Basic information of the participants is collected through questionnaire surveys and physical examination data collection, and fasting blood samples of the subjects are collected after a three-day (72 h) sampling period. The whole blood samples were centrifuged at 3,000 rpm for 10 min immediately after collection and the plasma samples were kept at −80°C until laboratory analysis. All blood samples were collected by professional medical staff. After excluding subjects with invalid personal PM_2.5_ samples or missing information, a total of 112 subjects were included in the final analysis. The current research was approved by the Ethics Committee of Fuzhou Center for Disease Control and Prevention (no.2021004). All participants provided informed consent before the survey.

### 2.2 Personal 24-h PM_2.5_ and PM_2.5_-bound PAHs monitoring

All participants wore Ultrasonic Personal Air Sample (UPAS V2, Access Sensor Technologies, CO, USA) at a flow rate of 1 L/min for a 72-h period to assess individual PM_2.5_ concentrations_._ The 24-h personal PM_2.5_ and PM_2.5_-bound PAHs mass concentrations were determined following standard operation procedures. The details of these procedures can be found in previous studies (10, 11, 14). In brief, we collected the filters from the samplers after sampling, and measure the mass concentration of PM_2.5_ by gravimetric method, and one half of each filter was used to measure PAH concentrations using high performance liquid chromatograph. In this study, we measured 16 PAHs designated as high-priority pollutants by the United States Environmental Protection Agency and two were excluded because more than 90% of the samples did not reach the detection limit. Specifically, 14 PAHs including Naphthalene (Nap), Acenaphthene (Ace), Fluorene (Flu), Phenanthrene (Phe), Fluoranthene (Flt), Pyrene (Pyr), Chrysene (Chr), Benz[a]anthracene (BaA), Benzo[b]fluoranthene (BbF), Benzo[k]fluoranthene (BkF), Benzo[a]pyrene (Bap), Dibenz[a,h]anthracene (Daha), Benzo[g,h,i]perylene (Bghip) and Indeno[1,2,3-cd]pyrene (Icdp).

The 14 included PAHs were divided according to their rings and molecular weight (1): 2rings (Nap); 3rings (Ace, Flu, Phe); 4rings (Flt, Pyr, Chr, BaA,); 5rings (BbF, BkF, Bap, Daha); 6rings (Bghip, Icdp) (2); low-molecular-weight PAHs (LMW-PAHs), including those with fewer than four rings, (Nap, Ace, Flu, Phe); high-molecular-weight PAHs (HMW-PAHs), including those with four or more rings.

### 2.3 Non-target metabolite detection and identification

Each sample was processed using Agilent 1,260 Infinity II coupled with a single quadrupole LC/MSD system. Plasma samples were separated on an Agilent SB-C18 column (2.1 × 50 mm, 1.8 μm, Agilent Technologies, Santa Clara, CA, USA) with an injection volume of 20 μL at a flow rate of 0.25 mL/min and a column temperature of 37°. These analytical methods for plasma untargeted metabolomics were commonly used in our previous studies.

Data files were converted to the mzXML format using Agilent MassHunter qualitative analysis software (version B.01.00, Agilent Technologies, Santa Clara, CA, USA) and processed using XCMS online (https://xcmsonline.scripps.edu, accessed on 29 September 2022), which performed feature detection, retention time correction, alignment, annotation, etc. We aligned the detected peaks across all samples with a retention time tolerance of 30 s and MS tolerance of 0.025 Da, and aligned peaks with a detection rate exceeding 80% were picked as potential metabolite features. Identification of metabolites was carried out by matching m/z (Da) values against the Human Metabolome Database (www.hmdb.ca, accessed on 10 October 2022), Kyoto Encyclopedia of Genes and Genomes (www.genome.jp/kegg/, accessed on 15 October 2022) and Metabolite Link (metlin.scripps.edu, accessed on 20 October 2022). Before statistical analysis, each metabolic peak in all subject samples was normalized based on QC samples.

### 2.4 Statistical analysis

LC–MS raw data, exported from XCMS Online, underwent preprocessing including baseline filtering, peak identification, peak integration, retention time (RT) correction, peak alignment, and normalization. The data was processed to generate a data matrix containing retention time, mass-to-charge ratio (m/z), peak intensity, and sample information. The peak intensity of metabolites in each sample was corrected using quality control (QC) sample peak intensities and subsequently normalized, we add one injection of QC at the beginning and end of each batch, and then normalize each batch based on the QC values from that batch to reduce batch effects. Metabolic features were matched against public databases (HMDB: www.hmdb.ca; KEGG: www.genome.jp/kegg/) using their exact mass, isotopic pattern, and MS/MS spectra. Matching criteria included an exact mass tolerance of 25 ppm and a retention time (RT) tolerance of ±30 s. When a metabolic feature matched multiple MS/MS spectra, all matching spectra were utilized for identification. Metabolite identification required meeting three stringent criteria (1): a defined RT window tolerance (2), an exact mass error tolerance of less than 10 ppm, and (3) a matching MS/MS spectrum based on comparison of ions present in the experimental spectrum with those in the database library entry.

We firstly examined the normality of data by Shapiro–Wilk test. Inhaled doses of PM_2.5_ and PM_2.5_-bound PAHs were log-transformed before further analyzing. We used a general linear regression model to estimate the association between the 14 PM_2.5_-bound PAHs and the log transformed peak areas of the metabolite features measured in plasma. Similar to some other metabolomics researches in air pollution, we adjusted all analyses for age, BMI, gender, season, smoking and drinking status (32, 33). Metabolite pathway enrichment analysis was performed by MetaboAnalyst (www.metaboanalyst.ca, accessed on 25 December 2022) according to the KEGG pathway database. Enrichment factor (EF) refers to a quantitative indicator that evaluates the effectiveness of enrichment. It reflects the ratio between the proportion of metabolites in a specific pathway within a selected group and their proportion in the background metabolites. Generally, a higher enrichment factor indicates stronger enrichment efficacy and greater importance of the metabolic pathway. The calculation formula is as follows:


$$\text{enrichment factor}=\frac{m/M}{n/N}$$

Where:

m: Number of metabolites in the target pathway within the selected group.

M: Number of metabolites in the same pathway within the background reference.

n: Total number of metabolites in the selected group.

N: Total number of background metabolites.

R 4.2.0 was used to complete the above analysis. All *p* values were based on the bilateral test, and the statistical test level was *α* = 0.05. Draw volcano maps of differential metabolites.

## 3 Results

### 3.1 Study sample characteristics

The demographic characteristics of the participants in baseline are presented in Table 1. Of the 112 participants, 69 (61.6%) were women. The average age and BMI of the subjects were 67.43 ± 5.98 years old and 23.17 ± 2.84 kg/m^3^, respectively. Other general information, such as the education and cooking information of the old people, are shown in Table 1.

**Table 1:** Demographic characteristics of 112 participants.

Element	Total (*N* = 112)	Summer (*N* = 56)	Winter (*N* = 56)	*p* ^a^
Age (years)	67.43 ± 5.98	67.98 ± 6.18	66.88 ± 5.78	0.330
BMI (kg/m^2^)	23.17 ± 2.84	23.11 ± 4.08	23.63 ± 2.78	0.433
Indoor time (h)	20.17 ± 3.55	20.34 ± 4.22	20.00 ± 2.74	0.615
Outdoor time (h)	3.70 ± 2.70	3.27 ± 2.64	4.13 ± 2.70	0.092
Gender				
Female	69 (61.6)	33 (58.9)	36 (64.3)	0.349
Male	43 (38.4)	23 (41.4)	20 (35.7)	
Education				
High School or lower	35 (31.2)	33 (58.9)	36 (64.3)	0.560
Higher education	34 (30.4)	23 (41.4)	20 (35.7)	
Smoking				
No	96 (85.7)	46 (82.1)	50 (89.3)	0.280
Yes	16 (14.3)	10 (17.9)	6 (10.7)	
Drinking				
No	80 (71.4)	43 (76.8)	37 (66.1)	0.209
Yes	32 (28.6)	13 (23.2)	19 (33.9)	
Exercises per week				
0	12 (10.7)	7 (12.5)	5 (8.9)	0.541
1–3	15 (13.4)	9 (16.1)	6 (10.7)	
>3	85 (75.9)	40 (71.4)	45 (80.4)	
Incense burning				
No	72 (64.3)	30 (53.6)	42 (75.0)	0.018*
Yes	40 (35.7)	26 (46.4)	14 (25.0)	
T (°C)	23.16 ± 6.40	29.13 ± 2.65	17.18 ± 1.67	<0.001***
Rhum (%)	76.52 ± 11.42	80.07 ± 10.75	72.96 ± 11.03	0.001**
Cooking				
No	37 (33.0)	8 (14.3)	29 (51.8)	<0.001***
Yes	75 (67.0)	48 (85.7)	27 (48.2)	
Cooking Fuel				
Coal Gas	35 (31.3)	17 (30.4)	18 (32.1)	0.838
Other	77 (68.8)	39 (69.6)	28 (67.9)	
Air Purifiers				
No	105 (93.8)	53 (94.6)	52 (92.9)	0.480
Yes	7 (6.3)	3 (5.4)	4 (7.1)	

Results are presented as Mean ± Standard Deviation or as a number (n) and percentage (%).

a Analysis of variance (ANOVA) was used to compare differences between seasons for continuous variables, while the chi-square test was employed for categorical variables to assess differences between seasons. Bold values are used to highlight values <0.05. *Represents *p*-value less than 0.05 but greater than 0.001, **represents *p*-value equal to 0.001, ***represents p less than 0.001.

### 3.2 The distributions of personal PM_2.5_ and PM_2.5_-bound PAHs

**Figure 1:**
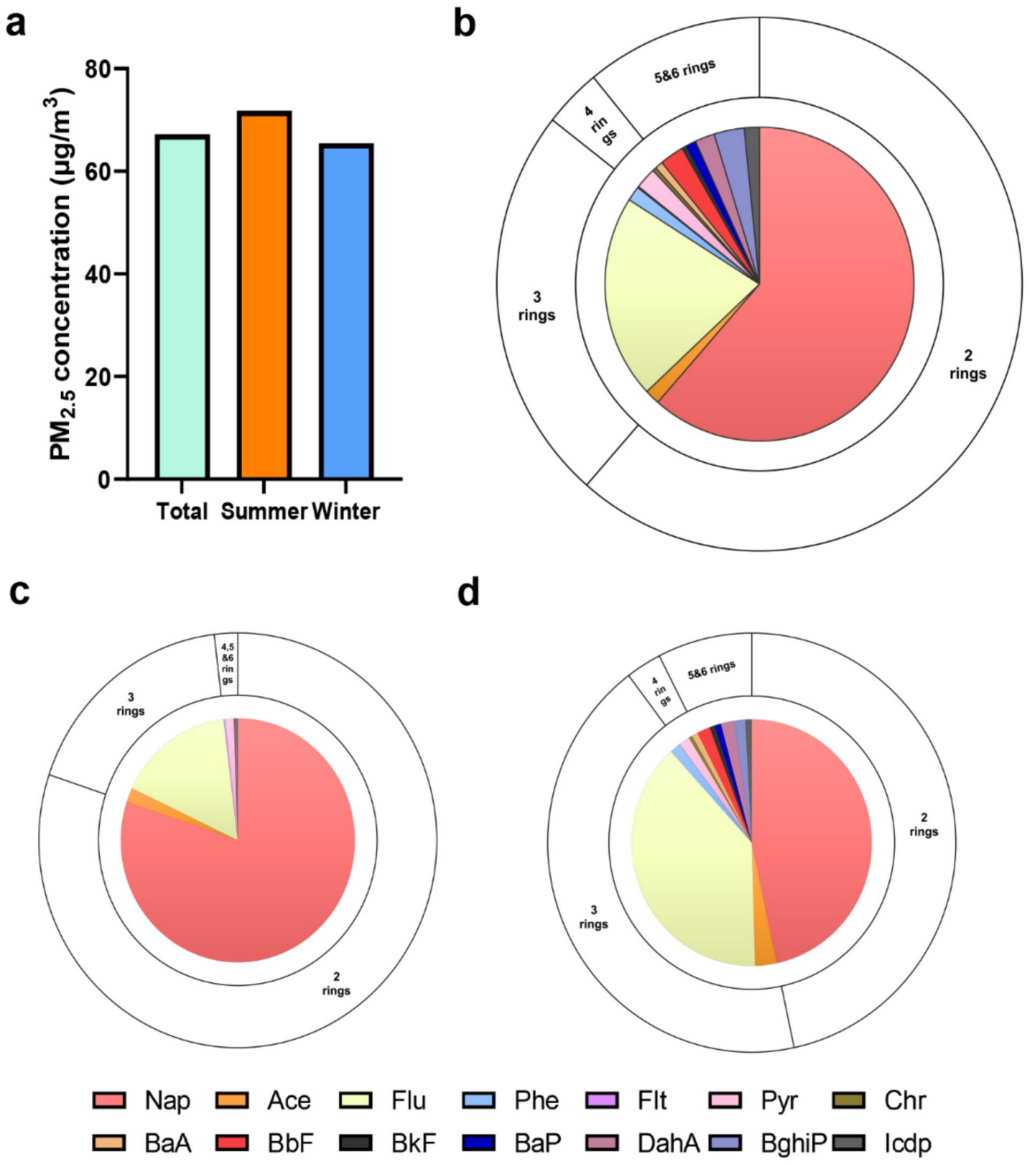
Descriptive distribution of the personal PM_2.5_ and PM_2.5_-bound PAHs. **(a)** The concentrations of personal PM_2.5_ in total visit, summer and winter. The concentrations and compositional distribution of PM_2.5_-bound PAHs in total visit **(b)**, summer **(c)** and winter **(d)**.

The median PM_2.5_ concentration across all observations was recorded at 69.4 ± 29.4 μg/m^3^. Summer and winter concentrations measuring at 75.1 ± 26.8 μg/m^3^ and 63.7 ± 30.9 μg/m^3^, respectively (Figure 1a). The distribution of the 14 PAHs is depicted in Figure 1b, and we have observed that the low molecular weight PAHs consisting of two and three rings account for the majority of PAHs at 14.215 ng/m^3^ (61%), and 5.581 ng/m^3^ (24%), respectively, while the high molecular weight PAHs with four to six rings only constitute 3.344 ng/m^3^ (15%). We also found significant differences in the concentration and composition of PM_2.5_-bound PAHs to which individuals were exposed in summer and winter (Table 2). From Figures 1c,d, we can see that there are significant differences in the composition of PAHs in different seasons. HMW PAHs show a more pronounced accumulation in winter, while low molecular weight PAHs account for a larger proportion in both summer and winter. This may be associated with the inherent temperature and humidity differences of the seasons, changes in behavior patterns brought about by the seasons, and the cooking conditions and energy sources of the subjects under study.

**Table 2:** Seasonal stratification results of individual PM_2.5_ bound PAHs.

	Summer (*N* = 56)	Winter (*N* = 56)	*p* ^a^
Nap	14.968 (1.955, 23.928)	22.354 (0.035, 44.264)	0.797
Ace	0.361 (0.178, 1.724)	1.584 (0.254, 3.371)	0.002**
Flu	2.601(1.574, 7.084)	22.241 (2.023, 38.774)	<0.001***
Phe	0.010 (0.010, 0.362)	0.808 (0.101, 1.119)	<0.001***
Flt	0.030 (0.030, 1.022)	0.370 (0.030, 0.978)	0.123
Pyr	0.267 (0.092, 0.620)	0.703 (0.301, 6.507)	<0.001***
Chr	0.089 (0.005, 0.192)	0.174 (0.005, 0.996)	0.113
BaA	0.010 (0.010, 0.185)	0.434 (0.184, 0.634)	<0.001***
BbF	0.010 (0.010, 0.582)	1.012 (0.527, 2.317)	<0.001***
BkF	0.005 (0.005, 0.005)	0.369 (0.093, 0.953)	<0.001***
BaP	0.005 (0.005, 0.093)	0.461 (0.342, 1.125)	<0.001***
DahA	0.010 (0.010, 0.454)	1.049 (0.487, 1.754)	<0.001***
BghiP	0.025 (0.025, 0.695)	0.831 (0.025, 1.449)	<0.001***
Icdp	0.025 (0.025, 0.025)	0.400 (0.025, 0.784)	<0.001***
LMW PAHs	19.410 (4.808, 33.245)	50.107 (2.977, 88.597)	0.111
HMW PAHs	2.101 (1.112, 3.670)	10.731 (5.204, 17.253)	<0.001***
2rings PAHs	14.968 (1.955, 23.928)	22.354 (0.035, 44.264)	0.797
3rings PAHs	4.153 (2.253, 7.257)	27.593 (2.939, 42.209)	<0.001***
4rings PAHs	0.854 (0.401, 1.982)	2.842 (1.050, 8.542)	<0.001***
5rings PAHs	0.438 (0.030, 1.205)	3.328 (2.939, 42.209)	<0.001***
6rings PAHs	0.050 (0.050, 0.759)	1.353 (0.050, 2.529)	0.001**
Total PAHs	22.332 (9.096, 35.717)	61.387 (21.666, 96.351)	<0.001***

a Mann–Whitney U test was used to compare differences between seasons.

*p* < 0.05. Bold values are used to highlight values < 0.05. *Represents *p*-value less than 0.05 but greater than 0.001, **represents *p*-value equal to 0.001, ***represents *p* less than 0.001.

### 3.3 Analysis of factors influencing personal PM_2.5_ and PM_2.5_-bound PAHs

Multiple linear regression modeling was conducted to investigate factors influencing individual-level PM_2.5_ and its bound PAHs. Using a stepwise method, variables with statistically significant differences (*p* < 0.05) were included in the model. The variables encompassed age, gender, BMI, daily time spent at home, ambient temperature and humidity, smoking status (yes vs. no), alcohol consumption (yes vs. no), cooking activity (yes vs. no), coal use (yes vs. no), air purifier usage (yes vs. no), indoor incense burning (yes vs. no), education level (above high school vs. high school or below), and dry cleaning frequency (more than twice weekly vs. twice or fewer weekly). All categorical variables were coded with the latter category as the reference group (Table 3). The study revealed that seasons significantly influence the composition of PM_2.5_-bound PAHs. Concentrations of all PAH types were higher in winter compared to summer, with particularly pronounced seasonal differences observed for 2–3 ring PAHs, which were elevated by 13.833 and 31.554 units, respectively. For 4–6 ring PAHs, the increases were 1.699, 3.347, and 1.797 units, respectively. These findings highlight seasonality as a critical factor affecting PAH levels in individual PM_2.5_ exposure. Additionally, individual PM_2.5_ exposure levels showed positive and significant associations with temperature, summer season, and cleaning frequency (more than twice weekly). Specifically, higher PM_2.5_ levels were observed in summer than in winter, and individuals performing dry cleaning more than twice weekly exhibited elevated PM_2.5_ exposure. Notably, older adults engaged in kitchen cooking demonstrated increased concentrations of 2–3 ring PAHs (*β* = 21.662 and 24.986, respectively), while their exposure to 4–5 ring PAHs decreased (*β* = −3.397 and −1.328). No significant association was found with 6-ring PAHs.

**Table 3:** The multivariate regression analysis of the impact on individual PM_2.5_ and PAHs component exposure levels.

Variable	*β*	*S_β_*	*t*	*p*
PM_2.5_				
Season (Summer vs. Winter)	19.423	0.332	3.562	0.001**
Temperature (°C)	0.905	0.197	2.296	0.024*
Cleaning/week (>2 vs. ≤ 2)	13.178	0.185	2.163	0.033*
2rings PAHs				
Cooking (Yes vs. No)	21.662	0.347	3.520	0.001**
Season (Summer vs. Winter)	−13.833	−0.235	−2.390	0.019*
3rings PAHs				
Season (Summer vs. Winter)	−31.554	−0.607	−7.106	<0.001***
Cooking (Yes vs. No)	24.986	0.452	5.242	<0.001***
Gender (Male vs. Female)	8.999	0.168	2.125	0.036*
4rings PAHs				
Cooking (Yes vs. No)	−3.397	−0.391	−4.358	<0.001***
Season (Summer vs. Winter)	−1.699	−0.208	−2.318	0.022*
5rings PAHs				
Season (Summer vs. Winter)	−3.347	−0.491	−6.493	<0.001***
Rhum (%)	−0.077	−0.257	−3.671	<0.001***
Cooking (Yes vs. No)	−1.328	−0.183	−2.412	0.018*
Age (years)	−0.121	−0.212	−2.992	0.003**
Gender (Male vs. Female)	1.322	0.189	2.668	0.009**
6rings PAHs				
Season (Summer vs. Winter)	−1.797	−0.320	−3.547	0.001**

The latter is the reference group, *p* < 0.05. Bold values are used to highlight values < 0.05. *Represents *p*-value less than 0.05 but greater than 0.001, **represents *p*-value equal to 0.001, ***represents p less than 0.001.

### 3.4 Metabolic dysfunction in older adults identifies molecular signatures and pathways linked to PM_2.5_-bound PAHs

#### 3.4.1 Differential metabolite screening

**Figure 2:**
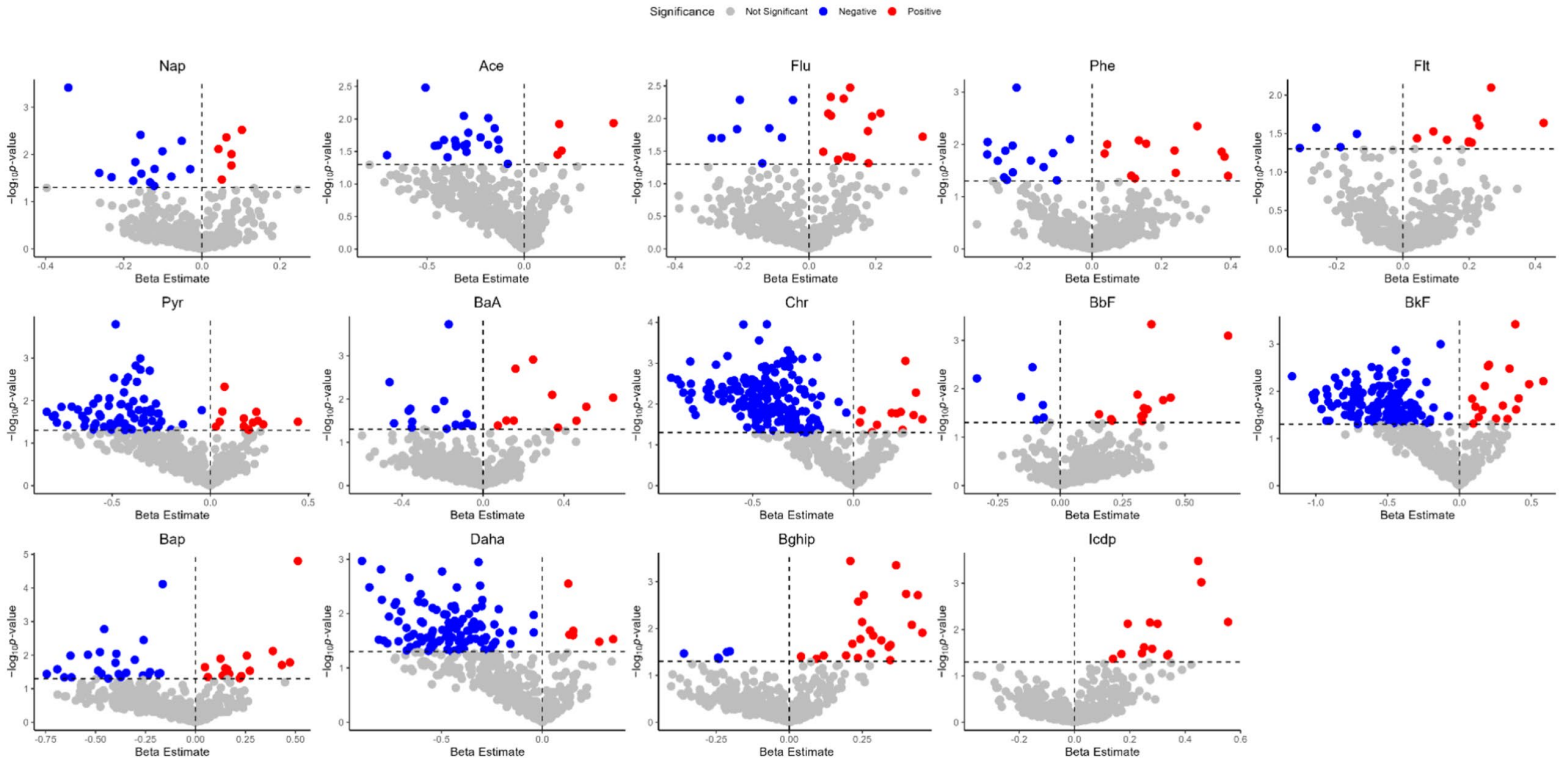
Volcano plots of the beta estimates of the PM_2.5_-bound PAHs vs. the *p* values in the associations of the exposure–plasma metabolome in total visit (*p* < 0.05).

As a first step, we examined the association between plasma metabolites and concentration of 14 PAHs in 112 samples. The numbers and directions of the associations between the PAHs are presented in Figure 2 (Red dots indicate positively correlated metabolites, blue indicates negatively correlated metabolites, and gray indicates no significant metabolites). This study found that the number of metabolites associated with different PAHs varies, with 4-5-ring PAHs generally affecting the most metabolites, especially chr; while 2–3 ring and 6-ring PAHs have fewer plasma metabolites affected. The specific list of metabolites and statistical data for the 14 PAHs can be found in the attached table.

#### 3.4.2 Metabolite pathway enrichment analysis

**Figure 3:**
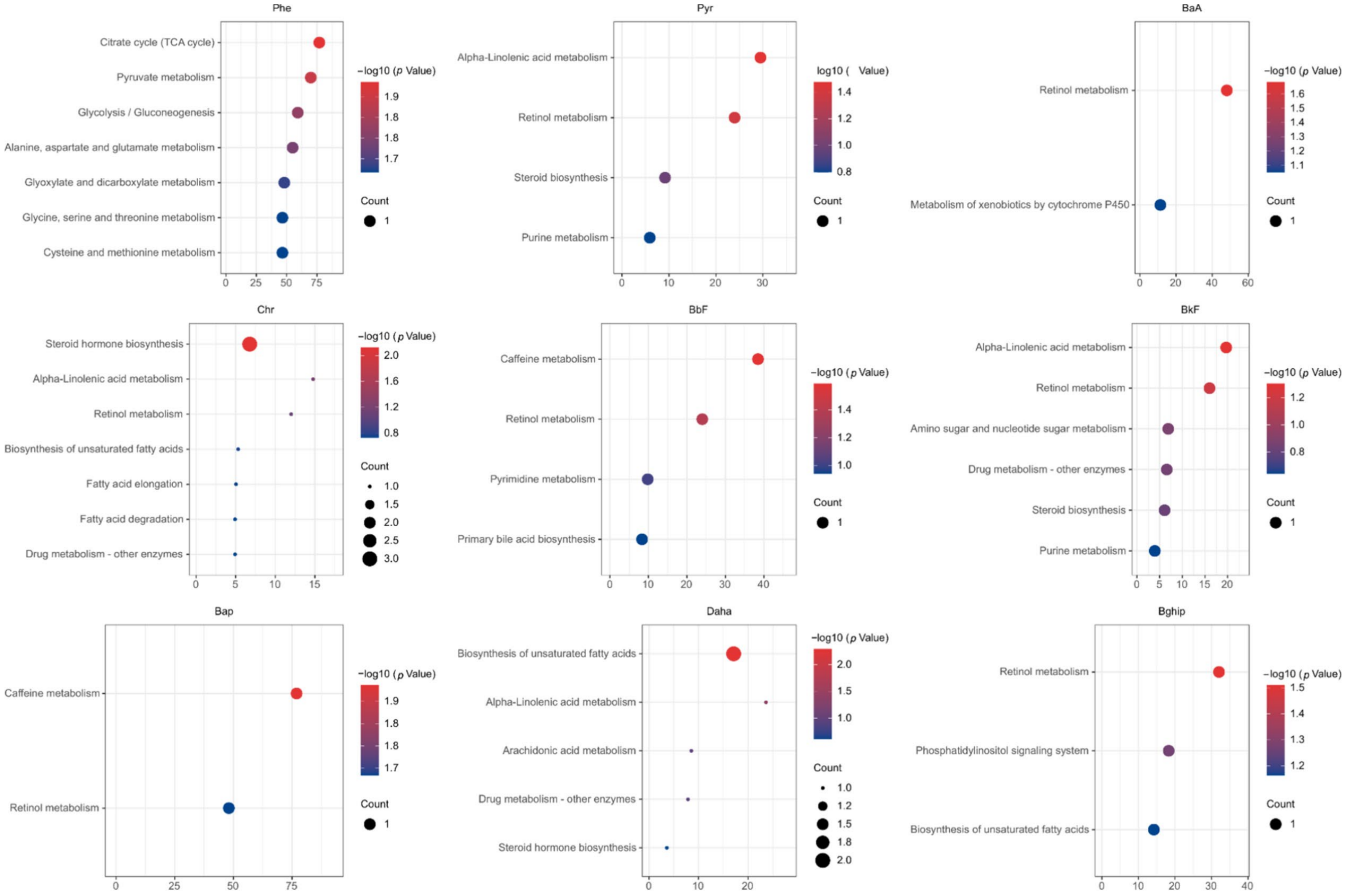
The bubble chart shows the results of the matched pathways enriched by the KEGG library according to *p*-values from pathway enrichment analysis and pathway impact values (x-axis) from pathway topology analysis (*p* < 0.05).

**Figure 4:**
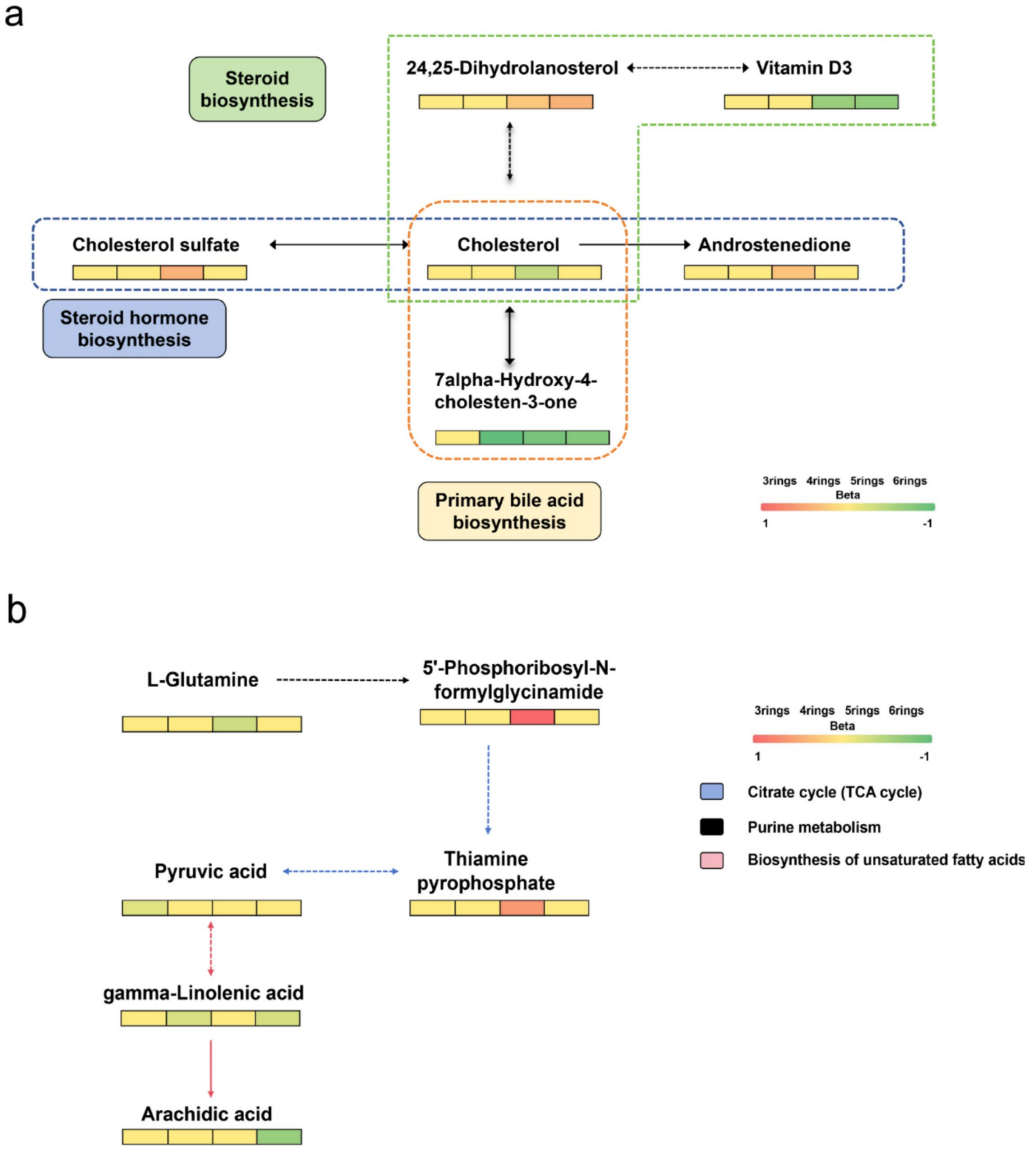
Heatmap of differential metabolic pathways for PAHs carried by PM_2.5_. **(a)** Metabolites involved in pathways of cholesterol biosynthesis, cholesterol hormone biosynthesis, and primary bile acid synthesis. **(b)** Metabolites involved in pathways of pyrimidine metabolism, citrate cycle, and unsaturated fatty acid biosynthesis.

**Figure 5:**
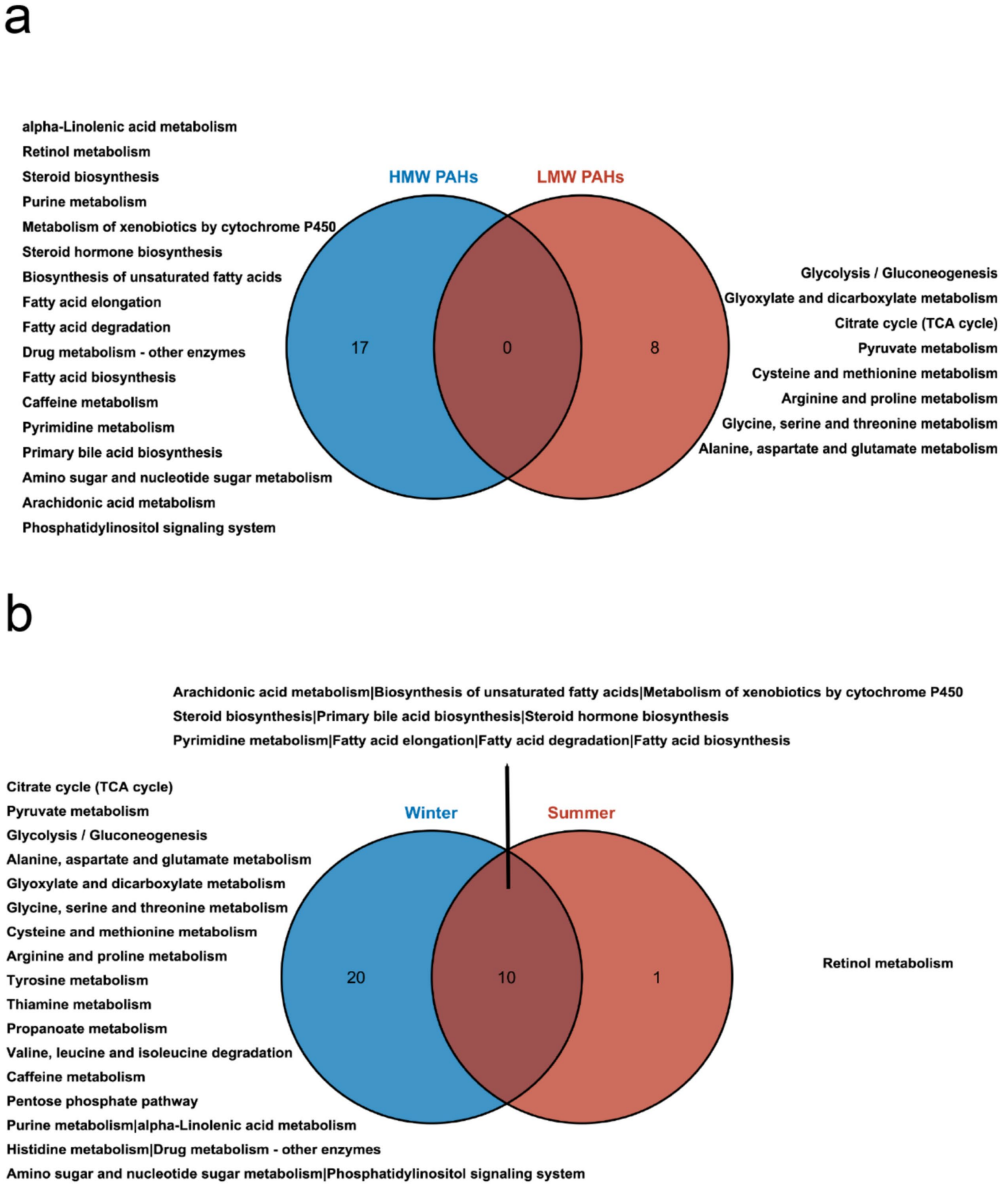
Comparative analysis of metabolic pathways influenced by PAHs in PM_2.5_. **(a)** Venn diagram illustrating the differential pathways influenced by high molecular weight and low molecular weight PAHs. **(b)** Venn diagram depicting the differences in pathways influenced by PAHs in summer and winter.

We next searched for the most significant metabolic pathways affected by PM_2.5_-bound PAHs through pathways enrichment analysis. The enrichment method is used by hypergeometric test while the topology analysis is used by relative-betweenness centrality. Enrichment analysis exhibited that the disturbed metabolites in plasma take part in kinds of biological pathways. Generally, a higher enrichment factor indicates stronger enrichment efficacy and greater importance of the metabolic pathway (Figure 3). We found impacts on metabolic pathways in 9 types of PAHs, including but not limited to amino acid metabolism, energy metabolism, carbohydrate metabolism and lipid metabolism. However, no enriched metabolic pathways were found for Daha, Nap, BaA, Flu and Flt PAHs. Our study further revealed that metabolites associated with PM₂.₅-bound PAH exposure were significantly enriched in bile acid metabolism and glutathione metabolism pathways. These findings indicate that PAH components may contribute to PM₂.₅-induced hepatic injury by disrupting amino acid metabolism and interfering with bile acid synthesis (Table 4). We generated heatmaps of the metabolic pathways of PAHs with different ring numbers, and the results suggest that the cholesterol and citric acid metabolites, as well as the cholesterol biosynthesis and citric acid cycle pathways they affect, may play important roles in the health damage caused by PAHs. The pathways with upstream-downstream relationships can be mainly divided into two parts: one includes cholesterol biosynthesis, cholesteryl hormone biosynthesis, and primary bile acid biosynthesis pathways; the other consists of pyrimidine metabolism, citric acid cycle, and unsaturated fatty acid biosynthesis pathways (Figure 4). To further clarify the effects of different PAHs on metabolites and metabolic pathways, differences in metabolic pathways affected by high molecular weight and low molecular weight PAHs were explored, and we found that exposure to LMW PAHs in PM_2.5_ were correlated with amino acid metabolic pathways, while HMW-PAHs are associated with fatty acid and cholesterol metabolism pathways (Figure 5a).

**Table 4:** Metabolic enrichment pathways related to PAHs exposure.

Pathway	Hits	*p*-value	Enrichment ratio
Total visit			
Ace (3 rings)			
Retinol metabolism	Jan-17	0.0258	45.249
Metabolism of xenobiotics by cytochrome P450	Jan-68	0.0864	11.312
Phe (3 rings)			
Citrate cycle (TCA cycle)	Jan-20	0.0258	38.462
Pyruvate metabolism	Jan-23	0.0297	33.445
Glycolysis/Gluconeogenesis	Jan-26	0.0335	29.586
Alanine, aspartate and glutamate metabolism	Jan-28	0.0361	27.473
Lipoic acid metabolism	Jan-28	0.0361	27.473
Glyoxylate and dicarboxylate metabolism	Jan-31	0.0399	24.814
Glycine, serine and threonine metabolism	Jan-33	0.0424	23.31
Cysteine and methionine metabolism	Jan-33	0.0424	23.31
Arginine and proline metabolism	Jan-36	0.0463	21.368
Pyrimidine metabolism	Jan-39	0.0501	19.724
Tyrosine metabolism	Jan-42	0.0539	18.315
Pyr (4 rings)			
Biosynthesis of unsaturated fatty acids	Jan-36	0.0463	29.499
Primary bile acid biosynthesis	Jan-46	0.0589	16.722
Chr (4 rings)			
Lysine degradation	Feb-30	0.033	6.849
Steroid hormone biosynthesis	Mar-87	0.0485	3.538
Nitrogen metabolism	01-Jun	0.0572	17.094
Steroid biosynthesis	Feb-41	0.0584	5
Thiamine metabolism	01-Jul	0.0664	14.663
Primary bile acid biosynthesis	Feb-46	0.0717	4.464
alpha-Linolenic acid metabolism	Jan-13	0.12	7.874
Arginine biosynthesis	Jan-14	0.129	7.353
Histidine metabolism	Jan-16	0.146	6.41
Purine metabolism	Feb-70	0.146	2.933
Retinol metabolism	Jan-17	0.154	6.024
Citrate cycle (TCA cycle)	Jan-20	0.179	5.128
Propanoate metabolism	Jan-21	0.187	4.878
Pyruvate metabolism	Jan-23	0.203	4.464
Glycolysis/Gluconeogenesis	Jan-26	0.226	3.953
Alanine, aspartate and glutamate metabolism	Jan-28	0.242	3.663
Lipoic acid metabolism	Jan-28	0.242	3.311
Glyoxylate and dicarboxylate metabolism	Jan-31	0.264	2.849
Biosynthesis of unsaturated fatty acids	Jan-36	0.3	2.632
Pyrimidine metabolism	Jan-39	0.321	2.632
Valine, leucine and isoleucine degradation	Jan-39	0.321	2.632
Drug metabolism - other enzymes	Jan-39	0.321	2.632
Metabolism of xenobiotics by cytochrome P450	Jan-68	0.494	1.508
BbF (5 rings)			
Retinol metabolism	Jan-17	0.022	45.249
Primary bile acid biosynthesis	Jan-46	0.0589	16.722
BkF (5 rings)			
Retinol metabolism	Jan-17	0.0435	22.624
Drug metabolism - other enzymes	Jan-39	0.0977	9.901
Steroid biosynthesis	Jan-41	0.102	9.346
Primary bile acid biosynthesis	Jan-46	0.114	8.333
Bap (5rings)			
Retinol metabolism	Jan-17	0.0328	30.211
Amino sugar and nucleotide sugar metabolism	Jan-42	0.0797	12.21
Primary bile acid biosynthesis	Jan-46	0.0871	11.148
Bghip (6 rings)			
Biosynthesis of unsaturated fatty acids	Feb-36	0.0104	12.195
Histidine metabolism	Jan-16	0.0707	13.736
Retinol metabolism	Jan-17	0.0749	12.937
Steroid biosynthesis	Jan-41	0.173	5.376
Amino sugar and nucleotide sugar metabolism	Jan-42	0.176	5.236
Primary bile acid biosynthesis	Jan-46	0.192	4.785
Icdp (6 rings)			
Biosynthesis of unsaturated fatty acids	Jan-36	0.0686	14.245
Steroid biosynthesis	Jan-41	0.0779	12.516
Primary bile acid biosynthesis	Jan-46	0.0871	11.148
Summer visit			
Flu			
Steroid biosynthesis	Jan-41	0.0266	37.594
Primary bile acid biosynthesis	Jan-46	0.0299	33.445
Steroid hormone biosynthesis	Jan-87	0.0565	17.699
Flt			
Citrate cycle (TCA cycle)	Jan-20	0.013	76.923
Pyruvate metabolism	Jan-23	0.0149	67.114
Glycolysis/Gluconeogenesis	Jan-26	0.0169	59.172
Alanine, aspartate and glutamate metabolism	Jan-28	0.0182	54.945
Lipoic acid metabolism	Jan-28	0.0182	54.945
Glyoxylate and dicarboxylate metabolism	Jan-31	0.0201	49.751
Glycine, serine and threonine metabolism	Jan-33	0.0214	46.729
Cysteine and methionine metabolism	Jan-33	0.0214	46.729
Arginine and proline metabolism	Jan-36	0.0234	42.735
Tyrosine metabolism	Jan-42	0.0273	36.63
BaA			
Thiamine metabolism	01-Jul	0.00908	109.89
Retinol metabolism	Jan-17	0.022	45.249
Citrate cycle (TCA cycle)	Jan-20	0.0258	38.462
Propanoate metabolism	Jan-21	0.0271	36.63
Pyruvate metabolism	Jan-23	0.0297	33.445
Glycolysis/Gluconeogenesis	Jan-26	0.0335	29.586
Lipoic acid metabolism	Jan-28	0.0361	27.473
Lysine degradation	Jan-30	0.0386	25.641
Valine, leucine and isoleucine degradation	Jan-39	0.0501	19.724
Bap			
Retinol metabolism	01-Jul	0.022	45.249
Biosynthesis of unsaturated fatty acids	Jan-36	0.0463	21.368
Bghip			
Biosynthesis of unsaturated fatty acids	Jan-36	0.0234	42.735
Arachidonic acid metabolism	Jan-44	0.0286	34.965
Icdp			
Biosynthesis of unsaturated fatty acids	Jan-36	0.0234	42.735
Arachidonic acid metabolism	Jan-44	0.0286	34.965
Winter visit			
Nap			
Primary bile acid biosynthesis	Feb-46	0.00505	16.667
Thiamine metabolism	01-Jul	0.0181	54.945
Citrate cycle (TCA cycle)	Jan-20	0.051	19.231
Propanoate metabolism	Jan-21	0.0535	18.315
Pyruvate metabolism	Jan-23	0.0585	16.722
Glycolysis/Gluconeogenesis	Jan-26	0.0659	14.793
Lipoic acid metabolism	Jan-28	0.0709	13.736
Lysine degradation	Jan-30	0.0758	12.821
Biosynthesis of unsaturated fatty acids	Jan-36	0.0904	10.684
Valine, leucine and isoleucine degradation	Jan-39	0.0977	9.901
Steroid biosynthesis	Jan-41	0.102	9.346
Steroid hormone biosynthesis	Jan-87	0.208	4.425
Ace			
Thiamine metabolism	01-Jul	0.0136	73.529
Citrate cycle (TCA cycle)	Jan-20	0.0385	25.641
Propanoate metabolism	Jan-21	0.0404	24.45
Pyruvate metabolism	Jan-23	0.0442	22.321
Glycolysis/Gluconeogenesis	Jan-26	0.0499	19.724
Lipoic acid metabolism	Jan-28	0.0536	18.315
Lysine degradation	Jan-30	0.0574	17.094
Ace			
Valine, leucine and isoleucine degradation	Jan-39	0.0742	13.158
Primary bile acid biosynthesis	Jan-46	0.0871	11.148
Metabolism of xenobiotics by cytochrome P450	Jan-68	0.127	7.519
Flu			
Primary bile acid biosynthesis	Feb-46	0.00257	22.297
Steroid biosynthesis	Jan-41	0.0779	12.516
Metabolism of xenobiotics by cytochrome P450	Jan-68	0.127	7.519
Steroid hormone biosynthesis	Jan-87	0.16	5.882
Phe			
Citrate cycle (TCA cycle)	Jan-20	0.013	76.923
Pyruvate metabolism	Jan-23	0.0149	67.114
Glycolysis/Gluconeogenesis	Jan-26	0.0169	59.172
Alanine, aspartate and glutamate metabolism	Jan-28	0.0182	54.945
Lipoic acid metabolism	Jan-28	0.0182	54.945
Glyoxylate and dicarboxylate metabolism	Jan-31	0.0201	49.751
Glycine, serine and threonine metabolism	Jan-33	0.0214	46.729
Cysteine and methionine metabolism	Jan-33	0.0214	46.729
Arginine and proline metabolism	Jan-36	0.0234	42.735
Tyrosine metabolism	Jan-42	0.0273	36.63
Pyr			
Biosynthesis of unsaturated fatty acids	Feb-36	0.0031	21.368
Retinol metabolism	Jan-17	0.0435	22.624
Arachidonic acid metabolism	Jan-44	0.11	8.772
BaA			
Androstenedione Metabolism	Jan-24	0.0474	20.877
Androgen and Estrogen Metabolism	Jan-33	0.0648	15.175
Steroid Biosynthesis	Jan-48	0.0936	10.438
Chr			
Lysine degradation	Feb-30	0.0215	8.547
Nitrogen metabolism	01-Jun	0.046	21.368
Thiamine metabolism	01-Jul	0.0534	18.315
alpha-Linolenic acid metabolism	Jan-13	0.0971	9.901
Purine metabolism	Feb-70	0.1	3.663
Arginine biosynthesis	Jan-14	0.104	9.174
Histidine metabolism	Jan-16	0.118	8
Retinol metabolism	Jan-17	0.125	7.519
Ubiquinone and other terpenoid-quinone biosynthesis	Jan-18	0.132	7.143
Citrate cycle (TCA cycle)	Jan-20	0.146	6.41
Propanoate metabolism	Jan-21	0.153	6.098
Pyruvate metabolism	Jan-23	0.166	5.587
Glycolysis/Gluconeogenesis	Jan-26	0.186	4.926
Alanine, aspartate and glutamate metabolism	Jan-28	0.198	4.587
Lipoic acid metabolism	Jan-28	0.198	4.587
Glyoxylate and dicarboxylate metabolism	Jan-31	0.217	4.132
Biosynthesis of unsaturated fatty acids	Jan-36	0.248	3.559
Pyrimidine metabolism	Jan-39	0.266	3.289
Valine, leucine and isoleucine degradation	Jan-39	0.266	3.289
Drug metabolism - other enzymes	Jan-39	0.266	3.289
Primary bile acid biosynthesis	Jan-46	0.306	2.786
Steroid hormone biosynthesis	Jan-87	0.504	1.475
BkF			
Primary bile acid biosynthesis	Feb-46	0.0167	9.569
Nitrogen metabolism	01-Jun	0.027	36.63
Alpha-Linolenic acid metabolism	Jan-13	0.0578	16.92
Arginine biosynthesis	Jan-14	0.0621	15.699
Histidine metabolism	Jan-16	0.0707	13.736
Alanine, aspartate and glutamate metabolism	Jan-28	0.121	7.874
Glyoxylate and dicarboxylate metabolism	Jan-31	0.133	7.092
Biosynthesis of unsaturated fatty acids	Jan-36	0.153	6.098
Pyrimidine metabolism	Jan-39	0.165	5.65
Drug metabolism - other enzymes	Jan-39	0.165	5.65
Purine metabolism	Jan-70	0.279	3.145
Bghip			
Biosynthesis of unsaturated fatty acids	Mar-36	0.000386	18.293
Thiamine metabolism	01-Jul	0.0315	31.447
Citrate cycle (TCA cycle)	Jan-20	0.0877	10.989
Propanoate metabolism	Jan-21	0.0919	10.471
Pyruvate metabolism	Jan-23	0.1	9.524
Glycolysis/Gluconeogenesis	Jan-26	0.113	8.475
Lipoic acid metabolism	Jan-28	0.121	7.874
Lysine degradation	Jan-30	0.129	7.353
Valine, leucine and isoleucine degradation	Jan-39	0.165	5.65
Steroid biosynthesis	Jan-41	0.173	5.376
Amino sugar and nucleotide sugar metabolism	Jan-42	0.176	5.236
Arachidonic acid metabolism	Jan-44	0.184	5
Primary bile acid biosynthesis	Jan-46	0.192	4.785
Icdp			
Biosynthesis of unsaturated fatty acids	Feb-36	0.0031	21.368
Steroid biosynthesis	Jan-31	0.102	9.346
Arachidonic acid metabolism	Jan-41	0.11	8.772
Primary bile acid biosynthesis	Jan-46	0.114	8.333

Due to significant variations in the composition and concentration of PAHs across different seasons, we further conducted a subgroup analysis based on seasons to uncover differences in the effects of PAHs on metabolites across different seasons. To further explore the type of biological responses induced by PM_2.5_ samples collected in different seasons, we compared the metabolic pathways in summer and winter (Figure 5b). The results revealed that PAHs had a broader and stronger impact on pathways during the winter season. Both seasons exhibited effects on cholesterol and fatty acid metabolism pathways. Unique metabolic pathways during the summer season included retinol metabolism. In contrast, the winter season predominantly featured pathways related to carbohydrate metabolism, amino acid metabolism, and energy metabolism.

## 4 Discussion

In this study conducted among older adults in Fuzhou, significant differences in plasma metabolic profiles over short-term exposure to PM_2.5_-bound PAHs were observed. The significantly altered metabolites were involved in metabolic pathways such as amino acid metabolism, energy metabolism, carbohydrate metabolism and lipid metabolism. To the best of our knowledge, this study firstly clarified the metabolic changes in plasma of individuals exposed to PAHs based on untargeted metabolomic approach in conjunction with the older adult study.

It has been well-recognized that different emission resources result in different types of atmospheric PAHs. Considering the differential health effects of various types of PAHs, we investigated their unique mechanisms of action based on molecular weight. LMW-PAHs most often come from natural resources (petrogenic) and HMW-PAHs often originate from combustion (pyrolytic), such as vehicle exhausts, wood combustion, oil burning (10, 11). This study elucidates that distinct categories of PAH mixtures induce significant, yet differential alterations in metabolic pathways. We observed that Nap and Flu were the most abundant compounds in the total PAHs. Despite their higher concentrations, compared to other high molecular weight PAHs, their impact on metabolic pathways is relatively minor. Among the LMW PAHs, only Phe was identified to be enriched in metabolic pathways implicated in glutathione metabolism, energy metabolism, and carbohydrate metabolism. This observation aligns with the findings of Jiang et al. (34). Glutathione, known for its role in regulating the oxidative-reductive state, is considered integral to the biological effects of Phe exposure due to its impact on antioxidant status. The citric acid cycle, the final common oxidative pathway for carbohydrates, fats, and amino acids, is pivotal for energy supply to the body. The Tricarboxylic Acid (TCA) cycle serves as a central pathway, connecting nearly all individual metabolic pathways (35). Citrate, an endogenous metabolite generated from glucose metabolism and fatty acid, is regulated by glucose levels and insulin (36). The TCA cycle and pyruvate metabolism are implicated in the pathogenesis of diabetes (37, 38), and citrate has been associated with kidney injury (39). Thus, PAHs may exacerbate kidney injury and diabetes by influencing energy and carbohydrate metabolism. Intriguingly, exposure to HMW PAHs significantly altered lipid metabolism, including fatty acid, alpha-linolenic acid metabolism, arachidonic acid metabolism, and cholesterol metabolism pathways. Several studies have demonstrated that lipid peroxidation and lipid-related dysfunctions are induced by PAH exposure in humans (40–42). Certain unsaturated fatty acids, such as linoleic acid, are reported to regulate host metabolism and possess anticarcinogenic properties (43). Moreover, the metabolism of arachidonic acid is believed to significantly influence the onset and progression of liver cancer (44). Schulze et al. (45) reported that free fatty acids are involved in energy generation, and up-regulation of cardiac lipid metabolism occurs during heart failure. Bile acids, critical signaling molecules that control hepatocellular function, and glutathione, an important antioxidant that protects the liver against oxidative injury, are also implicated (46, 47). Some research suggests that LysoPCs and LysoPEs play critical roles in PM exposure, potentially leading to vascular endothelial dysfunction. LysoPC (48) and LysoPE (49) are considered potential biomarkers for cardiovascular diseases (50, 51). However, the specific mechanism of lipid metabolism disturbance following PM_2.5_ exposure remains to be explored. PM_2.5_ can damage the cardiovascular system through mechanisms such as oxidative stress and inflammatory responses, and impair glucose homeostasis by affecting insulin response in the liver and adipose tissue (52). This subsequently leads to dysfunction in lipid metabolism (53, 54). Therefore, adverse lipid metabolism and lipid oxidation induced by inflammation may represent a potential mechanistic link between air pollution and dysregulated lipid levels (55, 56). This study hypothesizes that PM_2.5_ pollutants may influence the onset and progression of diabetes and cardiovascular diseases through the aforementioned metabolic pathways. This study also found that the metabolic pathways affected by PM_2.5_-bound PAHs encompass amino acid metabolism, carbohydrate metabolism, energy metabolism, and lipid-related metabolic processes. Specifically, they impact unsaturated fatty acid metabolism, cholesterol-related metabolic pathways, and upstream/downstream metabolites of the citrate cycle. Two longitudinal studies have identified a significant association between PAHs in PM_2.5_ and blood lipid profiles (10, 11). PAHs may contribute to PM_2.5_-induced liver damage by affecting amino acid metabolism and bile acid synthesis. In this study, we discovered that PM_2.5_-bound HMW PAHs interfere with lipid metabolism by mediating biosynthetic pathways related to fatty acids, steroids, bile acids, linoleic acid, and arachidonic acid. This further implicates their involvement in the alteration of lipid profiles and pathogenic processes associated with PM_2.5_ exposure. A metabolomic study on mice (50) exposure to PM_2.5_ and another study on rats (57) both observed the significant alterations of amino acid metabolism, lipid metabolism and glucose metabolism, consistent with the current research. These findings underscore the complex dynamics of PM_2.5_-bound PAHs and their potential health implications.

In this study, we further stratified the data by season to investigate the effects of seasonal pollutants on plasma metabolites and potential biological mechanisms. A total of 21 metabolic pathways were identified to exhibit differential expression between summer and winter. Notably, PAHs in winter induced a greater number of metabolic pathways, leading to more potent biological effects. Our results revealed that PM_2.5_ pollution in winter was more toxic than in summer, consistent with the levels of air pollution. A pivotal finding from our analyses was the identification of several biological pathways associated with elevated air pollution. Many of these identified pathways could precipitate subsequent consequences such as oxidative stress and inflammation, and are also implicated in coronary artery disease and atherosclerosis, as well as major adverse cardiovascular events, including myocardial infarction, stroke, and even death. These findings underscore the complex dynamics of seasonal air pollution and its potential health implications.

The strength of this study lies in its use of a community-based air pollution monitoring project involving older adults. This approach allowed for an exploration and correlation analysis of short-term exposure to PM_2.5_-bound PAHs with plasma metabolites. Another notable strength is the accurate assessment of an individual’s actual exposure using a personal exposure sampler. In epidemiological studies on air pollution, personal monitoring is deemed the gold standard for measuring actual individual exposure. However, there are some shortcomings: due to high costs and significant participant burden, personal monitoring is less frequently employed; emphasizes the limited sample scope (single community, small older adult cohort) and its potential geographical bias; Highlights the diverse pollution sources of PAHs bound to PM_2.5_ and their impact on exposure assessment accuracy. This study allows for a more accurate understanding of exposure through individual air monitoring in the older adult sensitive population, and enables a more meticulous establishment of biological connections.

Looking ahead, further validation of these results can be achieved by increasing the sample size and extending the study duration. Future investigations will necessitate the assessment of the toxicological significance of these pathways by establishing their specific associations with injury. Given that many oxidation and nitration products of polycyclic aromatic hydrocarbons are considered harmful to the human body, additional research could explore the health effects of other byproducts, apart from the parent compounds. These considerations provide a roadmap for future research, underscoring the importance of continued exploration in this critical area of public health.

## 5 Conclusion

The metabolomic signatures discerned in this study could potentially elucidate the effects of PM_2.5_-bound PAHs on energy metabolism, carbohydrate metabolism, and lipid metabolism disruption. The findings of this investigation indicate that different components of PAHs influence health outcomes in varying ways and provide potential mechanisms underlying the pathogenic processes of PAHs in PM_2.5_. The identified biomarker panels and metabolic pathways could serve as preventive targets for addressing diseases associated with PM_2.5_-bound PAHs exposure. These insights offer a novel perspective on understanding the health implications of PM_2.5_ pollution on susceptible older adult populations, providing potential biological underpinnings. This not only enriches our knowledge in this field but also paves the way for future research.

## Data Availability

The original contributions presented in the study are included in the article/Supplementary material, further inquiries can be directed to the corresponding authors.
