# Supplementary material for: Metabolome-wide associations with short-term exposure to PM2.5-bound polycyclic aromatic hydrocarbons: a study in older adults
Source: Front Public Health. 2025 Jul 24;13:1609724. doi: 10.3389/fpubh.2025.1609724 (PMC12328383; doi:10.3389/fpubh.2025.1609724)
Supplement: Supplementary file 1 [file Data_Sheet_1.docx]

Appendix

TableA1 Metabolite data

| Number | Metabolite |
| --- | --- |
| M001 | C33H68 |
| M002 | C21H36NO |
| M003 | C29H53NO5 |
| M004 | C16H12Cl2O4 |
| M005 | C31H50O5 |
| M006 | C19H38O |
| M007 | C40H77NO3 |
| M008 | C9H14Br2O2 |
| M009 | C66H120O6 |
| M010 | C69H122O6 |
| M011 | C13H24O3 |
| M012 | C69H125O33P |
| M013 | C10H17N3O6 |
| M014 | C47H82O2 |
| M015 | C39H81N2O6P |
| M016 | C18H39N3O |
| M017 | C17H14Cl2O4 |
| M018 | C30H58O4S |
| M020 | C46H82NO9P |
| M021 | C30H61NO3 |
| M022 | C52H80O2 |
| M023 | C8H15N2O9P |
| M024 | C58H110O14 |
| M026 | C31H41N7O6 |
| M027 | C52H94O6 |
| M029 | C48H96NO6P |
| M030 | C23H48O |
| M033 | C30H51NO6 |
| M034 | C15H32N2 |
| M035 | C47H84O2 |
| M036 | C31H40O2 |
| M037 | C48H52O26 |
| M038 | C23H44 |
| M039 | C23H48O2 |
| M041 | C24H36O2 |
| M045 | C23H48O |
| M047 | C15H30O2 |
| M050 | C35H67NO4 |
| M051 | C41H32O27 |

TableA1 Metabolite data（continued）

| Number | Metabolite |
| --- | --- |
| M052 | C27H44NO7P |
| M053 | C31H64O |
| M055 | C22H38O2 |
| M056 | C59H118NO8P |
| M057 | C47H93N2O6P |
| M058 | C35H53NO3 |
| M062 | C48H82NO7P |
| M065 | C36H74O2 |
| M066 | C20H44N |
| M067 | C36H74O2 |
| M068 | C30H60NO7P |
| M069 | C15H27NO4 |
| M070 | C21H27NO2 |
| M072 | C45H80O |
| M074 | C36H74O2 |
| M075 | C40H80O2 |
| M077 | C128H236O16 |
| M078 | C20H41NO4S |
| M079 | C19H42NO5P |
| M080 | C27H48N2 |
| M082 | C57H104O9 |
| M083 | C15H13NO2 |
| M084 | C40H80NO8P |
| M085 | C30H60O |
| M088 | C24H34O2 |
| M089 | C90H154N4O42 |
| M091 | C46H79NO8 |
| M092 | C19H39O7P |
| M093 | C19H38O2 |
| M094 | C20H42O2 |
| M095 | C49H78O2 |
| M097 | C20H40 |
| M098 | C35H45NO11 |
| M099 | C33H54O2 |
| M100 | C25H52O2 |
| M101 | C16H30O4 |
| M102 | C17H14N2O7S3 |
| M103 | C27H37D7O2 |
| M104 | C17H32O |
| M105 | C29H50O2 |
| M106 | C49H86NO13P |
| M107 | Tomatidine |

TableA1 Metabolite data（continued）

| Number | Metabolite |
| --- | --- |
| M108 | C27H41D3O3 |
| M109 | C40H54O |
| M110 | C20H24D8O2 |
| M111 | TG(i-19:0/i-20:0/19:0) |
| M113 | C47H92NO7P |
| M114 | C8H18N2O3S |
| M115 | C19H41N3O |
| M116 | C45H82O5 |
| M119 | C24H40O4 |
| M120 | C35H74N |
| M122 | C21H42NO2 |
| M123 | C35H56 |
| M124 | C56H109NO4 |
| M125 | C31H34O10 |
| M126 | C24H38O12 |
| M127 | C24H48O2 |
| M128 | C15H25NO4 |
| M129 | C47H96N2O6P |
| M130 | C49H96O5 |
| M131 | C25H48O4 |
| M133 | C19H30O3 |
| M134 | C36H74O2 |
| M135 | C16H32O2 |
| M136 | C27H54O2 |
| M137 | C48H77N15O11 |
| M138 | C5H10N2O |
| M139 | C17H22N2 |
| M140 | C44H89NO4 |
| M141 | C24H42 |
| M142 | C12H23N |
| M143 | C20H33NO |
| M144 | C15H20O5 |
| M145 | C15H28O4 |
| M146 | C11H18O |
| M147 | C19H35NO2 |
| M148 | C32H54O4 |
| M149 | C27H54O4 |
| M150 | C20H37NO3 |
| M151 | C29H52NO12P |
| M152 | C28H50O2 |
| M154 | C27H37ClN2O4 |
| M156 | C13H24O3 |

TableA1 Metabolite data（continued）

| Number | Metabolite |
| --- | --- |
| M157 | C15H27D3O2 |
| M159 | C11H12N2OS |
| M161 | C19H32O4 |
| M162 | C66H110O6 |
| M163 | C8H4O12Sb2.2K.3H2O |
| M164 | C31H51N10O22P3S2 |
| M165 | C47H80O7 |
| M166 | C11H8O6 |
| M172 | C12H22N2 |
| M173 | C6H13N |
| M175 | C17H19NO3 |
| M176 | C27H50O2 |
| M179 | C5H13O14P3 |
| M180 | C47H88NO13P |
| M181 | C39H79N2O6P |
| M182 | C12H19N3O7S |
| M183 | C38H56O3 |
| M184 | C40H82NO7P |
| M185 | C41H58O2 |
| M186 | C23H40O |
| M187 | C13H28O4 |
| M190 | C27H43NO |
| M193 | C34H64O2 |
| M194 | C28H50O6 |
| M195 | C67H118O5 |
| M196 | C24H14D9NO2 |
| M197 | C19H28O2 |
| M198 | C18H34O4 |
| M200 | C32H54O6 |
| M201 | C33H66O3 |
| M203 | TG(i-19:0/19:0/i-22:0) |
| M204 | TG(24:1(15Z)/22:5(4Z,7Z,10Z,13Z,16Z)/22:6(4Z,7Z,10Z,13Z,16Z,19Z)) |
| M205 | trans-2-Hexacosenoic acid |
| M206 | C47H94NO8P |
| M209 | Taxane |
| M211 | Tandospirone |
| M212 | TG(15:0/20:4(8Z,11Z,14Z,17Z)/24:1(15Z)) |
| M213 | C18H36O3 |
| M214 | C38H68O5 |
| M215 | C27H46O2 |
| M216 | C50 H78 N O8 P |
| M217 | C16H32O |

TableA1 Metabolite data（continued）

| Number | Metabolite |
| --- | --- |
| M218 | C26H50O2 |
| M219 | C23H50NO6P |
| M220 | C10HCl11 |
| M221 | C28H46O4 |
| M222 | C23H48O |
| M226 | C26H45NO |
| M227 | TG(15:0/24:1(15Z)/18:0) |
| M228 | C18H36O3 |
| M230 | C33H64O2 |
| M231 | C82H164O4 |
| M232 | C52H104NO8P |
| M233 | C18H36O3 |
| M235 | C24H47NO4 |
| M236 | C36H60O2 |
| M237 | C30H50 |
| M238 | C28H58NO7P |
| M239 | C28H48O2 |
| M240 | C17H32O2 |
| M242 | C21H38O3 |
| M243 | C33H65NO3 |
| M244 | C20H36O6 |
| M245 | C20H38O5 |
| M246 | C22H37NO4 |
| M247 | C20H40O3 |
| M248 | C40H60 |
| M249 | C53H57O28 |
| M250 | C30H58NO7P |
| M252 | C36H60O9 |
| M253 | C18H39N |
| M254 | C15H10O13S2 |
| M255 | C31H52O3 |
| M256 | C24H38O2 |
| M257 | C47H84O2 |
| M258 | C9H17N3O4 |
| M259 | C9H10 |
| M260 | C46H60O7 |
| M261 | C6H13NO5 |
| M262 | C49H99N2O6P |
| M263 | C28H54O2 |
| M265 | C10H12N2O2 |
| M266 | C33H58O4 |
| M267 | C17H35NO |

TableA1 Metabolite data（continued）

| Number | Metabolite |
| --- | --- |
| M269 | C18H29N |
| M270 | C55H100O5 |
| M271 | C15H28 |
| M272 | C30H56O2 |
| M273 | C27H50O |
| M274 | C72H116O4 |
| M275 | C20H34O3 |
| M276 | C21H29N3O |
| M278 | C65H126O5 |
| M279 | C15H22N2O |
| M281 | C18H36O3 |
| M282 | C34H70O |
| M283 | C18H35NO3 |
| M284 | C40H64 |
| M285 | C21H44O3 |
| M286 | C29H58O |
| M287 | C57H104O5 |
| M288 | C17H32O2 |
| M289 | C9H21N2O3 |
| M290 | C43H84O5 |
| M291 | C29H56 |
| M292 | C10H22N4 |
| M293 | C39H68O2 |
| M294 | C33H64O2 |
| M295 | C25H46O |
| M296 | C30H52O |
| M297 | C18H36O3 |
| M298 | C26H46O5 |
| M299 | C18H37NO |
| M300 | C26H46N2O |
| M301 | C20H38 |
| M302 | C17H26N2O |
| M303 | C27H46O4S |
| M304 | C32H64NO7P |
| M305 | C32H38NO7 |
| M306 | C16H14O5 |
| M307 | C57H63O32 |
| M308 | C71H130O17P2 |
| M309 | C40H22O24 |
| M310 | C14H30O |
| M311 | 1-Phenyl-2-hexadecanoylamino-3-pyrrolidino-1-propanol |
| M314 | C10H15ClN5O12P3 |

TableA1 Metabolite data（continued）

| Number | Metabolite |
| --- | --- |
| M316 | C20H37D3O2 |
| M317 | C26H54O2 |
| M318 | C12H13NO2 |
| M319 | C9H14FN2O15P3 |
| M320 | C23H29NO9 |
| M321 | C30H37NO4 |
| M324 | 5-Pentacosyl-1,3-benzenediol |
| M325 | C23H48NO4P |
| M326 | C6H12O2 |
| M327 | C6H20O30P8 |
| M328 | C10H16 |
| M329 | Dihydroalprenolol |
| M330 | C19H35NO |
| M331 | C34H42N2O4 |
| M332 | C15H30O2 |
| M333 | C17H19N4O10P |
| M334 | TG(18:0/24:1(15Z)/22:4(7Z,10Z,13Z,16Z)) |
| M335 | C24H48O4 |
| M336 | C7H17N3 |
| M337 | C28H50 |
| M338 | C13H21NO6S |
| M339 | C15H21N |
| M340 | C10H20NO4 |
| M341 | C5H10N2O3 |
| M342 | C130H243N3O39P2 |
| M343 | C19H34O |
| M344 | C19H36O4 |
| M345 | C82H134O37 |
| M347 | C26H51NO3 |
| M348 | C28H48N2O4 |
| M349 | C50H94O6 |
| M350 | Labdane |
| M351 | C42H59NO5 |
| M352 | C22H24O13 |
| M353 | C27H31D6FO2 |
| M354 | C12H9ClO2 |
| M355 | C27H44O5 |
| M356 | C31H64O2 |
| M357 | C29H56O4 |
| M358 | C30H62NO7P |
| M359 | C36H73NO4 |
| M361 | C10H15N2O8P |

TableA1 Metabolite data（continued）

| Number | Metabolite |
| --- | --- |
| M362 | C47H97N2O6P |
| M363 | C39H56N7O21P3S |
| M364 | C15H27NO |
| M365 | C39H66O5 |
| M366 | C14H16N2O2 |
| M367 | C26H45NO2 |
| M368 | C23H38O3 |
| M369 | 8-Isoprostane |
| M370 | C12H19N4O7P2S |
| M371 | C18H28O2 |
| M372 | C19H26O2 |
| M373 | C20H42N6O5 |
| M374 | C39H56O2 |
| M375 | C38H76O2 |
| M376 | C42H82O4 |
| M377 | C51H59O32 |
| M378 | C16H16O11 |
| M379 | C5H9NO |
| M380 | C27H54NO7P |
| M381 | C49H80O2 |
| M382 | C18H30O3 |
| M383 | C18H30D4O4 |
| M384 | C28H48O |
| M385 | C77H146O17P2 |
| M386 | C33H56O4 |
| M388 | C10H18O5 |
| M389 | C19H24N2O8 |
| M391 | C15H30 |
| M392 | C42H41O26 |
| M393 | CE(14:1(9Z)) |
| M395 | C17H33NO4 |
| M396 | C35H60O2 |
| M397 | C26H47NO |
| M398 | C82H149N3O32 |
| M399 | C7H8N4O2 |
| M400 | C27H54O4 |
| M402 | Benzyl 2-methyl-3-oxobutanoate |
| M405 | 3-(2,4-Dichloro-5-methoxyphenyl)-2-sulfanyl-4(3H)-quinazolinone |
| M406 | Dechlorane plus |
| M407 | Muramic acid |
| M408 | LysoPA(20:2(11Z,14Z)/0:0) |
| M409 | oleanolic acid |

TableA1 Metabolite data（continued）

| Number | Metabolite |
| --- | --- |
| M410 | cis-erucic acid |
| M411 | Asparagoside B |
| M412 | Mycinamicin IV |
| M413 | Xylometazoline |
| M414 | C32H53NO |
| M415 | alpha-Tocopherol acetate |
| M416 | D-Threitol |
| M417 | 3-Hydroxyhexadecanoylcarnitine |
| M418 | Methylimidazoleacetic acid |
| M419 | Fabianine |
| M420 | Cyanidin 3-(6-coumaryl-2'-sinapoylsophoroside) 5-glucoside |
| M421 | 5,3'-Dihydroxy-6,7,4',5'-tetramethoxyflavanone |
| M422 | alpha-N-(3-octadecanoyloxy-octadecanoyl)-L-ornithine |
| M423 | Cer(d18:0/20:0) |
| M424 | 3-Epidemissidine |
| M425 | 1-O-eicosanoyl-Cer(d18:1/16:0) |
| M426 | xi-1-Butoxy-1-methoxyethane |
| M427 | Mestranol |
| M428 | 10,11-Dihydro-12R-hydroxy-leukotriene E4 |
| M429 | Ethylene glycol distearate |
| M430 | Dimethyldisulfide |
| M431 | 5-Chloro-1,3-dihydro-1-(4-piperidinyl)-2H-benzimidazol-2-one |
| M432 | C24 H50 N O8 P |
| M433 | 3-keto palmitic acid |
| M434 | Linoleic acid(d4) |
| M436 | 4-Hydroxy-5-(3',4'-dihydroxyphenyl)-valeric acid-O-glucuronide |
| M437 | KDNalpha2-3Galbeta1-4(Fucalpha1-3)GlcNAcbeta1-3Galbeta1-4Glcbeta-Cer(d18:1/24:1(15Z)) |
| M438 | S-Adenosyl-L-homocysteine |
| M440 | Tridecanamide |
| M441 | 2-eicosyl-3-hydroxy-heptatriaconta-16Z-enoic acid |
| M442 | Brassilexin |
| M443 | 3'-(beta-D-Glucopyranosyl)-2'-hydroxy-beta-oxodihydrochalcone |
| M444 | 3-Hydroxy-5Z-octenyl acetate |
| M445 | Bromobenzene |
| M446 | Trimethylaminoacetone |
| M447 | DG(20:3n9/0:0/20:2n6) |
| M448 | Ganglioside GT1b (d18:0/22:1(13Z)) |
| M449 | 1-O-(2-Acetamido-2-deoxy-alpha-D-glucopyranosyl)-1D-myo-inositol 3-phosphate |
| M450 | Trimethaphan |
| M452 | Pyruvic acid |
| M454 | Dalapon |

TableA1 Metabolite data（continued）

| Number | Metabolite |
| --- | --- |
| M455 | Lauric acid(d3) |
| M456 | 5'-Phosphoribosyl-N-formylglycinamide |
| M457 | Annonacin |
| M458 | Fludrocortisone acetate |
| M459 | Acamprosate |
| M460 | SM(d18:0/24:1(15Z)(OH)) |
| M461 | oleanolic acid |
| M462 | Dimethylarsinic acid |
| M463 | Sulfobromophthalein |
| M464 | N-(2-fluro-ethyl) arachidonoyl amine |
| M465 | Isoscoparine |
| M466 | Galacturonic acid |
| M467 | Avenanthramide C |
| M468 | Methoxyflurane |
| M469 | ortho-Vanadate |
| M470 | 3-Chloro-4-(dichloromethylene)-2,5-pyrrolidinedione |
| M471 | Trichloroethylene |
| M472 | 1,2-Di-(9Z,12Z-heptadecadienoyl)-3-(5Z,8Z,11Z,14Z-eicosatetraenoyl)-sn-glycerol |
| M473 | 2,2-dichloro-1,1-ethanediol |
| M474 | 2,4,5-Trichloro-2,5-cyclohexadiene-1-ol |
| M475 | Lisinopril |
| M476 | 1,2,3,4,7,8,9-Heptachlorodibenzofuran |
| M477 | Cer(d18:1/22:1(13Z)) |
| M478 | 22:1-Glc-Campesterol |
| M479 | Sodium chlorate |
| M480 | 2,3-Diketo-5-methylthio-1-phosphopentane |
| M481 | Phloretin |
| M482 | Erythrodiol 3-palmitate |
| M483 | Arachidic acid |
| M485 | 2-tetracosyl-3-hydroxy-32-methoxy-33-methyl-henpentacontanoic acid |
| M486 | 2-ethyl-1,5-dimethyl-3,3-diphenylpyrrolinium (EDDP) |
| M487 | 24-hydroxy-cholesterol(d6) |
| M488 | Cer(d15:2(4E,6E)/20:0(2OH)) |
| M489 | Vitamin D3 |
| M492 | 2-Amino-4-hydroxy-6-(D-erythro-1_2_3-trihydroxypropyl)-7_8-dihydropteridine |
| M493 | 5-Decanoyl-2-nonylpyridine |
| M496 | 2,5-Bis[(iodomercuri)methyl]-p-dioxane |
| M498 | DG(16:1(9Z)/0:0/16:1(9Z)) (d5) |
| M499 | Docosanamide |
| M500 | C6H15NO |
| M501 | C23H42 |
| M502 | C20H32O2 |

TableA1 Metabolite data（continued）

| Number | Metabolite |
| --- | --- |
| M503 | C15H21NO7 |
| M504 | C36H64O4 |
| M505 | C73H132O6 |
| M506 | C16H16ClNO3S |
| M508 | C44H89NO4 |
| M509 | C55H106O5 |
| M510 | C16H30O |
| M511 | C35H66O5 |
| M512 | C51H90O2 |
| M513 | C10H12BrCl2O3PS |
| M514 | C31H52O2 |
| M515 | C55H108O3 |
| M516 | C7H16O3 |
| M517 | C24H34O4 |
| M518 | C20H40O2 |
| M520 | C44H87NO3 |
| M521 | C33H58O5 |
| M523 | C19H38O2 |
| M524 | C71H114O6 |
| M525 | C25H52O2 |
| M526 | C37H67D5O5 |
| M527 | C50H88O |
| M528 | C10H19NO2 |
| M529 | C26H44O4 |
| M530 | C30H48O |
| M532 | C9H19N |
| M535 | C10H15NO |
| M536 | C12H17N |
| M537 | C35H66O4 |
| M538 | C27H44O7 |
| M539 | C11H12O5 |
| M540 | C27H53NO4 |
| M542 | C27H44O2 |
| M543 | C29H56O |
| M544 | C16H30O7 |
| M545 | C41H80O2 |
| M546 | C10H21NO2S |
| M547 | C21H32N2O2 |
| M548 | C24H40N2 |
| M549 | C51H91D5O6 |
| M550 | C29H50O |
| M551 | C26H45O11P |

TableA1 Metabolite data（continued）

| Number | Metabolite |
| --- | --- |
| M552 | C18H30 |
| M553 | C16H32O2 |
| M554 | C44H89NO4 |
| M555 | C20H32O2 |
| M556 | C25H49NO4S |
| M557 | C43H76O2 |
| M558 | C33H58O4 |
| M559 | C8H11N3O3 |
| M560 | C19H29NO3 |
| M561 | C36H68O2 |
| M562 | C34H64O6 |
| M563 | C34H65NO5 |
| M564 | C29H44O2 |
| M565 | C56H109NO4 |
| M567 | C71H118O6 |
| M568 | C27H53O12P |
| M569 | C37H74O4 |
| M570 | C22H42O3 |
| M571 | C6H20O30P8 |
| M572 | C59H115NO4 |
| M573 | C61H118O5 |
| M574 | C11H16 |
| M575 | C20H38O2 |
| M576 | C31H54O |
| M577 | C35H68O5 |
| M578 | C31H63NO3 |
| M580 | C18H30O2 |
| M581 | C20H28O |
| M582 | C21H24O11 |
| M583 | C34H60O4 |
| M584 | C27H37D7O2 |
| M585 | C23H44NO2 |
| M586 | C2H4 |
| M587 | C27H46O |
| M588 | C50H86O2 |
| M589 | C13H16O |
| M590 | C102H180N4O53 |
| M591 | C41H62O |
| M592 | C15H16N2O2 |
| M593 | C52H90O7 |
| M594 | C35H58O6 |
| M595 | C22H37NO5S |

TableA1 Metabolite data（continued）

| Number | Metabolite |
| --- | --- |
| M596 | C32H54O2 |
| M597 | C21H30O6 |
| M598 | C26H53NO2 |
| M599 | C29H46O2 |
| M600 | C40H79NO2 |
| M601 | C65H118O5 |
| M602 | C51H91D5O6 |
| M603 | C39H64O5 |
| M604 | C50H88O2 |
| M606 | C46H94NO7P |
| M607 | C2H3Br |
| M608 | C6FeN6 |
| M609 | C43H87NO5 |

TableB1 Metabolites related to Chr

| Metabolite | *p*-value |
| --- | --- |
| M560 | 0.00000617 |
| M611 | 0.0000983 |
| M616 | 0.000306183 |
| M300 | 0.000394428 |
| M270 | 0.000501768 |
| M602 | 0.000612011 |
| M282 | 0.00109492 |
| M039 | 0.001679435 |
| M539 | 0.001718577 |
| M100 | 0.001758203 |
| M010 | 0.001858653 |
| M151 | 0.001978707 |
| M463 | 0.00212964 |
| M077 | 0.002366329 |
| M553 | 0.002468455 |
| M260 | 0.002519274 |
| M146 | 0.003078396 |
| M409 | 0.003111542 |
| M302 | 0.003395599 |
| M244 | 0.003472282 |
| M604 | 0.00403107 |
| M274 | 0.004078732 |
| M144 | 0.004224414 |
| M355 | 0.004324243 |
| M291 | 0.004597385 |
| M094 | 0.004750549 |
| M030 | 0.004758262 |

TableB1 Metabolites related to Chr（continued）

| Metabolite | | *p*-value | |
| --- | --- | --- | --- |
| M527 | | 0.004763018 | |
| M162 | | 0.004789892 | |
| M593 | | 0.004857306 | |
| M023 | | 0.004903629 | |
| M212 | | 0.005014075 | |
| M033 | | 0.005018227 | |
| M056 | | 0.005103932 | |
| M026 | | 0.005185098 | |
| M536 | | 0.005205765 | |
| M001 | | 0.005520617 | |
| M015 | | 0.005594855 | |
| M122 | | 0.005626028 | |
| M123 | | 0.005644562 | |
| M446 | | 0.005795896 | |
| M247 | | 0.005934576 | |
| M205 | | 0.006056511 | |
| M411 | | 0.006328059 | |
| M319 | | 0.00637739 | |
| M257 | | 0.006483275 | |
| M347 | | 0.006868118 | |
| M047 | | 0.006893567 | |
| M080 | | 0.006956282 | |
| M326 | | 0.007331266 | |
| M058 | | 0.007394768 | |
| M243 | | 0.007426641 | |
| M598 | | 0.007481958 | |
| M017 | | 0.007519309 | |
| M055 | | 0.007840002 | |
| M083 | | 0.007861326 | |
| M003 | | 0.007983321 | |
| M193 | | 0.007991642 | |
| M066 | | 0.008149243 | |
| M556 | | 0.008299664 | |
| M198 | | 0.008362173 | |
| M009 | | 0.008388066 | |
| M148 | | 0.008891417 | |
| M203 | | 0.009095631 | |
| M293 | | 0.00927511 | |
| M187 | | 0.009366916 | |
| M600 | | 0.009406707 | |
| M505 | | 0.009641781 | |
| M034 | | 0.009735545 | |

TableB1 Metabolites related to Chr（continued）

| Metabolite | | *p*-value | |
| --- | --- | --- | --- |
| M427 | | 0.009779151 | |
| M053 | | 0.009835249 | |
| M141 | | 0.010230135 | |
| M599 | | 0.010599324 | |
| M231 | | 0.010644764 | |
| M402 | | 0.010708753 | |
| M101 | | 0.010711467 | |
| M314 | | 0.010828125 | |
| M084 | | 0.010830992 | |
| M105 | | 0.011157691 | |
| M217 | | 0.011353026 | |
| M085 | | 0.011380191 | |
| M070 | | 0.011408765 | |
| M292 | | 0.011487591 | |
| M289 | | 0.01184519 | |
| M371 | | 0.012075762 | |
| M062 | | 0.012109775 | |
| M052 | | 0.013006177 | |
| M013 | | 0.013073472 | |
| M067 | | 0.013235598 | |
| M249 | | 0.013252152 | |
| M008 | | 0.013361985 | |
| M271 | | 0.013431279 | |
| M107 | | 0.013738716 | |
| M429 | | 0.013750579 | |
| M334 | | 0.013857525 | |
| M395 | | 0.013882263 | |
| M163 | | 0.014046271 | |
| M156 | | 0.014111247 | |
| M227 | | 0.01412435 | |
| M521 | | 0.014439869 | |
| M255 | | 0.014906629 | |
| M455 | | 0.015077586 | |
| M154 | | 0.015404455 | |
| M104 | | 0.016233645 | |
| M221 | | 0.016525358 | |
| M114 | | 0.016682381 | |
| M197 | | 0.016993168 | |
| M172 | | 0.0171222 | |
| M020 | | 0.017396381 | |
| M145 | | 0.01740846 | |

TableB1 Metabolites related to Chr（continued）

| Metabolite | | *p*-value | |
| --- | --- | --- | --- |
| M021 | | 0.017434546 | |
| M358 | | 0.017665299 | |
| M176 | | 0.018130953 | |
| M142 | | 0.018925285 | |
| M149 | | 0.019523755 | |
| M605 | | 0.020203144 | |
| M252 | | 0.020245295 | |
| M152 | | 0.020256324 | |
| M339 | | 0.020258578 | |
| M130 | | 0.020716007 | |
| M098 | | 0.020760553 | |
| M356 | | 0.020796523 | |
| M147 | | 0.020810217 | |
| M594 | | 0.021090317 | |
| M245 | | 0.021191222 | |
| M547 | | 0.02142133 | |
| M194 | | 0.022081745 | |
| M037 | | 0.022177077 | |
| M380 | | 0.022860244 | |
| M109 | | 0.022906902 | |
| M459 | | 0.023265961 | |
| M200 | | 0.023320821 | |
| M365 | | 0.023341825 | |
| M201 | | 0.023432317 | |
| M011 | | 0.023812798 | |
| M186 | | 0.023966349 | |
| M139 | | 0.024032109 | |
| M308 | | 0.025026223 | |
| M006 | | 0.025243869 | |
| M213 | | 0.025527562 | |
| M108 | | 0.026083088 | |
| M175 | | 0.026400016 | |
| M418 | | 0.026425311 | |
| M431 | | 0.026638952 | |
| M219 | | 0.027136679 | |
| M324 | | 0.027406051 | |
| M089 | | 0.027744176 | |
| M357 | | 0.028045132 | |
| M353 | | 0.028655015 | |
| M136 | | 0.028935236 | |
| M041 | | 0.029071129 | |
| M372 | | 0.029754943 | |

TableB1 Metabolites related to Chr（continued）

| Metabolite | | *p*-value | |
| --- | --- | --- | --- |
| M321 | | 0.030064835 | |
| M016 | | 0.030388032 | |
| M518 | | 0.031397682 | |
| M183 | | 0.031604438 | |
| M330 | | 0.032773746 | |
| M385 | | 0.032924279 | |
| M180 | | 0.033392065 | |
| M038 | | 0.033425717 | |
| M412 | | 0.033780655 | |
| M004 | | 0.033906143 | |
| M097 | | 0.034134022 | |
| M432 | | 0.035093444 | |
| M079 | | 0.035099916 | |
| M440 | | 0.035362491 | |
| M574 | | 0.035590384 | |
| M386 | | 0.035826877 | |
| M029 | | 0.036379526 | |
| M354 | | 0.036386877 | |
| M075 | | 0.036390346 | |
| M024 | | 0.036548205 | |
| M329 | | 0.037671686 | |
| M317 | | 0.037793409 | |
| M133 | | 0.038048372 | |
| M296 | | 0.038756335 | |
| M370 | | 0.040248131 | |
| M209 | | 0.040965132 | |
| M609 | | 0.041199832 | |
| M126 | | 0.044206849 | |
| M434 | | 0.044225829 | |
| M327 | | 0.045112346 | |
| M582 | | 0.045735915 | |
| M577 | | 0.045870734 | |
| M391 | | 0.046064433 | |
| M072 | | 0.046285863 | |
| M088 | | 0.047027535 | |
| M248 | | 0.047220637 | |
| M341 | | 0.047780688 | |
| M303 | | 0.04801813 | |
| M420 | | 0.048060664 | |
| M398 | | 0.048523679 | |
| M364 | | 0.048772107 | |
| M471 | | 0.049453809 | |
| M159 | | 0.049505627 | |
| M111 | | 0.049963856 | |

TableB2 Metabolites related to icdp

| Metabolite | *p*-value |
| --- | --- |
| M555 | 0.00000286 |
| M510 | 0.00237653 |
| M611 | 0.004504556 |
| M621 | 0.008876541 |
| M593 | 0.011245273 |
| M592 | 0.01325683 |
| M596 | 0.01789342 |
| M340 | 0.019158325 |
| M590 | 0.020400204 |
| M556 | 0.022217575 |
| M560 | 0.024518198 |
| M530 | 0.026348461 |
| M512 | 0.028003618 |
| M591 | 0.028825306 |
| M566 | 0.032123129 |
| M546 | 0.034954142 |
| M587 | 0.035249781 |
| M574 | 0.036302684 |
| M567 | 0.03633858 |
| M568 | 0.036806283 |
| M296 | 0.038989922 |
| M598 | 0.047382299 |
| M468 | 0.049788899 |

TableB3 Metabolites related to ace

| Metabolite | *p*-value |
| --- | --- |
| M528 | 0.00072549 |
| M563 | 0.003102821 |
| M519 | 0.00744219 |
| M527 | 0.009236573 |
| M627 | 0.012718353 |
| M569 | 0.01336531 |
| M545 | 0.01426901 |
| M424 | 0.015997806 |
| M554 | 0.016180008 |
| M599 | 0.019869347 |
| M305 | 0.021797667 |
| M607 | 0.022452721 |
| M471 | 0.022852095 |
| M474 | 0.028190889 |
| M253 | 0.030973951 |
| M614 | 0.033059063 |
| M609 | 0.035385059 |
| M517 | 0.037623543 |
| M534 | 0.04045275 |
| M588 | 0.043228334 |
| M555 | 0.046220188 |

TableB4 Metabolites related to bap

| Metabolite | *p*-value |
| --- | --- |
| M527 | 0.000023 |
| M599 | 0.0000963 |
| M601 | 0.002556331 |
| M624 | 0.013860034 |
| M159 | 0.014189318 |
| M607 | 0.014712178 |
| M530 | 0.015329254 |
| M585 | 0.016287704 |
| M272 | 0.016945299 |
| M521 | 0.020162713 |
| M514 | 0.020345606 |
| M563 | 0.021423059 |
| M528 | 0.022549186 |
| M519 | 0.023163885 |
| M522 | 0.024145439 |
| M529 | 0.02501229 |
| M326 | 0.025403676 |
| M575 | 0.025964366 |
| M156 | 0.026180966 |
| M459 | 0.026553823 |
| M578 | 0.027329732 |
| M560 | 0.028117197 |
| M590 | 0.028726562 |
| M266 | 0.031178595 |
| M365 | 0.032041221 |
| M580 | 0.03422655 |
| M424 | 0.037900481 |
| M206 | 0.039480161 |
| M261 | 0.040480888 |
| M126 | 0.041482409 |
| M612 | 0.043035331 |
| M586 | 0.04703302 |
| M595 | 0.047067772 |
| M012 | 0.047171125 |

TableB5 Metabolites related to bbf

| Metabolite | *p*-value |
| --- | --- |
| M527 | 0.000650957 |
| M599 | 0.000662492 |
| M560 | 0.002495211 |
| M604 | 0.006369449 |
| M521 | 0.006656624 |
| M459 | 0.008357119 |
| M505 | 0.008474068 |
| M536 | 0.008757004 |
| M539 | 0.011977086 |
| M585 | 0.020088158 |
| M530 | 0.034840788 |
| M181 | 0.035533773 |
| M116 | 0.036364791 |
| M587 | 0.038299392 |
| M282 | 0.043808973 |
| M603 | 0.046163381 |
| M544 | 0.046827691 |
| M500 | 0.048754732 |
| M593 | 0.049083739 |

TableB6 Metabolites related to baa

| Metabolite | *p*-value |
| --- | --- |
| M527 | 0.00021767 |
| M599 | 0.000639728 |
| M424 | 0.002477374 |
| M607 | 0.002883976 |
| M459 | 0.003540046 |
| M553 | 0.004403137 |
| M514 | 0.004742304 |
| M467 | 0.009965749 |
| M308 | 0.011541081 |
| M521 | 0.011869286 |
| M270 | 0.012182259 |
| M588 | 0.012797627 |
| M511 | 0.015826003 |
| M624 | 0.016537756 |
| M265 | 0.019865381 |
| M612 | 0.019912519 |
| M595 | 0.021420341 |
| M528 | 0.024594329 |
| M619 | 0.026235791 |
| M603 | 0.026503453 |
| M311 | 0.026929801 |
| M247 | 0.02875708 |
| M618 | 0.030474577 |
| M529 | 0.030802793 |
| M231 | 0.034487475 |
| M505 | 0.037450974 |
| M556 | 0.047753816 |

TableB7 Metabolites related to flt

| Metabolite | *p*-value |
| --- | --- |
| M480 | 0.002211835 |
| M577 | 0.003028722 |
| M534 | 0.004281683 |
| M538 | 0.025671219 |
| M562 | 0.030616241 |
| M476 | 0.031152445 |
| M457 | 0.032992956 |
| M570 | 0.034490617 |
| M481 | 0.036399362 |
| M454 | 0.041198097 |
| M125 | 0.049711212 |

TableB8 Metabolites related to daha

| Metabolite | *p*-value |
| --- | --- |
| M448 | 0.004885842 |
| M514 | 0.008254713 |
| M427 | 0.009233238 |
| M271 | 0.009476052 |
| M228 | 0.010911203 |
| M527 | 0.011768911 |
| M603 | 0.014590793 |
| M077 | 0.016601975 |
| M303 | 0.016705673 |
| M129 | 0.018386638 |
| M376 | 0.019347309 |
| M354 | 0.020700238 |
| M440 | 0.021314535 |
| M425 | 0.022127987 |
| M596 | 0.023490383 |
| M351 | 0.024287494 |
| M181 | 0.026483738 |
| M209 | 0.02808169 |
| M566 | 0.031407409 |
| M337 | 0.032985877 |
| M163 | 0.033378025 |
| M095 | 0.034074508 |
| M262 | 0.035390622 |
| M141 | 0.035409632 |
| M107 | 0.03549842 |
| M072 | 0.036185655 |
| M423 | 0.038207002 |
| M624 | 0.038750862 |
| M529 | 0.041209954 |
| M139 | 0.041758674 |
| M227 | 0.041766099 |
| M226 | 0.043756812 |
| M197 | 0.044204306 |
| M056 | 0.045169527 |
| M285 | 0.045700301 |
| M335 | 0.047468007 |
| M350 | 0.049023927 |

TableB9 Metabolites related to bkf

| Metabolite | *p*-value |
| --- | --- |
| M527 | 0.0000181 |
| M599 | 0.0000246 |
| M560 | 0.000151673 |
| M521 | 0.000869766 |
| M553 | 0.001286216 |
| M528 | 0.001459491 |
| M603 | 0.002341037 |
| M159 | 0.002565333 |
| M539 | 0.002802233 |
| M600 | 0.003210278 |
| M556 | 0.004616691 |
| M500 | 0.004935044 |
| M505 | 0.005308049 |
| M511 | 0.005782297 |
| M282 | 0.005985682 |
| M590 | 0.007020224 |
| M588 | 0.007726163 |
| M514 | 0.007903763 |
| M265 | 0.008929605 |
| M607 | 0.009536769 |
| M326 | 0.010348993 |
| M530 | 0.010426492 |
| M459 | 0.010508793 |
| M077 | 0.010523322 |
| M567 | 0.011677111 |
| M058 | 0.012242964 |
| M010 | 0.012864057 |
| M039 | 0.016650456 |
| M305 | 0.017059056 |
| M574 | 0.017730443 |
| M398 | 0.017782031 |
| M609 | 0.018082733 |
| M429 | 0.018642415 |
| M012 | 0.019627556 |
| M270 | 0.020692739 |
| M148 | 0.022213228 |
| M156 | 0.023017935 |
| M299 | 0.024371044 |
| M554 | 0.02499083 |
| M388 | 0.025715153 |

TableB9 Metabolites related to bkf(continued)

| Metabolite | | *p*-value | |
| --- | --- | --- | --- |
| M163 | | 0.026371096 | |
| M274 | | 0.027608324 | |
| M200 | | 0.02762068 | |
| M004 | | 0.02767296 | |
| M613 | | 0.032541203 | |
| M193 | | 0.032913114 | |
| M099 | | 0.033203525 | |
| M424 | | 0.033840634 | |
| M314 | | 0.034975501 | |
| M056 | | 0.035248714 | |
| M190 | | 0.036702699 | |
| M070 | | 0.037437072 | |
| M409 | | 0.037800245 | |
| M227 | | 0.038456181 | |
| M319 | | 0.038563367 | |
| M008 | | 0.039377123 | |
| M018 | | 0.039893669 | |
| M120 | | 0.040252102 | |
| M238 | | 0.040279593 | |
| M100 | | 0.041034989 | |
| M529 | | 0.041520237 | |
| M507 | | 0.041969635 | |
| M141 | | 0.04230382 | |
| M463 | | 0.043228362 | |
| M041 | | 0.043685868 | |
| M197 | | 0.044590589 | |
| M231 | | 0.046283056 | |
| M149 | | 0.04683919 | |
| M151 | | 0.047370609 | |
| M016 | | 0.047644009 | |
| M015 | | 0.048734742 | |
| M259 | | 0.048859865 | |
| M052 | | 0.049707115 | |

TableB10 Metabolites related to flu

| Metabolite | *p*-value |
| --- | --- |
| M516 | 0.001135238 |
| M381 | 0.002677592 |
| M545 | 0.0033626 |
| M577 | 0.004284639 |
| M534 | 0.004492913 |
| M627 | 0.0051634 |
| M529 | 0.006257904 |
| M576 | 0.007139149 |
| M616 | 0.01079946 |
| M254 | 0.013463965 |
| M602 | 0.015299134 |
| M624 | 0.016852122 |
| M481 | 0.017597515 |
| M596 | 0.019610694 |
| M517 | 0.030844345 |
| M131 | 0.032470399 |
| M474 | 0.033169977 |
| M533 | 0.035866094 |
| M590 | 0.03916818 |
| M601 | 0.039230406 |
| M535 | 0.041302014 |
| M124 | 0.042178622 |
| M472 | 0.042462783 |
| M157 | 0.04410223 |
| M594 | 0.045689365 |
| M333 | 0.048506976 |

TableB11 Metabolites related to bghip

| Metabolite | *p*-value |
| --- | --- |
| M593 | 0.000143798 |
| M574 | 0.000324541 |
| M548 | 0.000340009 |
| M556 | 0.000567076 |
| M505 | 0.000585007 |
| M500 | 0.000591549 |
| M560 | 0.001026568 |
| M555 | 0.001209796 |
| M536 | 0.001762407 |
| M518 | 0.001935059 |
| M501 | 0.002236875 |
| M554 | 0.003330163 |
| M539 | 0.003581495 |
| M504 | 0.004160571 |
| M598 | 0.004398354 |
| M582 | 0.004452646 |
| M272 | 0.004900246 |
| M596 | 0.006376154 |
| M521 | 0.006754192 |
| M626 | 0.007781435 |
| M125 | 0.007857484 |
| M349 | 0.008188551 |
| M599 | 0.009466524 |
| M429 | 0.01237761 |
| M120 | 0.013176911 |
| M331 | 0.01452286 |
| M398 | 0.015065857 |
| M290 | 0.016479952 |
| M589 | 0.016499367 |
| M261 | 0.017825945 |
| M510 | 0.019734593 |
| M039 | 0.019740803 |
| M507 | 0.020300708 |
| M522 | 0.020461408 |
| M553 | 0.02109928 |
| M566 | 0.021371915 |
| M591 | 0.021613033 |
| M512 | 0.021885996 |
| M004 | 0.024627438 |
| M352 | 0.025190969 |
| M577 | 0.025285867 |
| M421 | 0.026210009 |

TableB11 Metabolites related to bghip(continued)

| Metabolite | | *p*-value | |
| --- | --- | --- | --- |
| M567 | | 0.026824466 | |
| M523 | | 0.02685306 | |
| M624 | | 0.029182224 | |
| M459 | | 0.029302101 | |
| M542 | | 0.029448254 | |
| M418 | | 0.029835836 | |
| M220 | | 0.030265978 | |
| M342 | | 0.032340728 | |
| M181 | | 0.034584563 | |
| M511 | | 0.034923925 | |
| M447 | | 0.03870956 | |
| M586 | | 0.040762362 | |
| M575 | | 0.041027472 | |
| M449 | | 0.041841496 | |
| M262 | | 0.041997495 | |
| M389 | | 0.04656564 | |
| M126 | | 0.0477617 | |
| M127 | | 0.047952215 | |
| M204 | | 0.049612044 | |

TableB12 Metabolites related to pyr

| Metabolite | *p*-value |
| --- | --- |
| M611 | 0.000022 |
| M594 | 0.00003 |
| M560 | 0.0000429 |
| M590 | 0.000107081 |
| M516 | 0.00137711 |
| M579 | 0.001893481 |
| M127 | 0.002104036 |
| M616 | 0.002802763 |
| M602 | 0.00337449 |
| M258 | 0.003602314 |
| M591 | 0.004568753 |
| M137 | 0.005583041 |
| M039 | 0.006229163 |
| M598 | 0.009588252 |
| M247 | 0.010170658 |
| M380 | 0.010513448 |
| M593 | 0.011583401 |
| M522 | 0.014033702 |
| M608 | 0.014554574 |
| M190 | 0.01832686 |
| M463 | 0.019248963 |
| M339 | 0.020584074 |
| M383 | 0.021163672 |
| M365 | 0.021582506 |
| M576 | 0.022265552 |
| M411 | 0.028070139 |
| M544 | 0.02922627 |
| M529 | 0.030002176 |
| M569 | 0.030206264 |
| M530 | 0.030870824 |
| M123 | 0.032660543 |
| M427 | 0.034045497 |
| M151 | 0.034901277 |
| M091 | 0.035316187 |
| M542 | 0.0358539 |
| M402 | 0.037298918 |
| M003 | 0.038043402 |
| M457 | 0.038408034 |
| M422 | 0.03912715 |
| M355 | 0.042748339 |
| M539 | 0.043697618 |
| M300 | 0.046878767 |
| M218 | 0.048933847 |
| M353 | 0.049274294 |

TableB13 Metabolites related to phe

| Metabolite | *p*-value |
| --- | --- |
| M535 | 0.002327297 |
| M569 | 0.003170338 |
| M591 | 0.005092209 |
| M297 | 0.012986167 |
| M316 | 0.013517137 |
| M457 | 0.017228787 |
| M184 | 0.022123761 |
| M310 | 0.022189435 |
| M612 | 0.022373684 |
| M452 | 0.02237648 |
| M479 | 0.023882068 |
| M383 | 0.025394511 |
| M131 | 0.028947445 |
| M617 | 0.03642688 |
| M361 | 0.037045167 |
| M546 | 0.038441917 |
| M095 | 0.040516747 |

TableB14 Metabolites related to nap

| Metabolite | *p*-value |
| --- | --- |
| M560 | 0.00123513 |
| M627 | 0.001285407 |
| M528 | 0.00130818 |
| M616 | 0.001430804 |
| M577 | 0.00210349 |
| M590 | 0.002122571 |
| M602 | 0.002443287 |
| M609 | 0.00444964 |
| M516 | 0.004751114 |
| M554 | 0.005209947 |
| M594 | 0.006552729 |
| M534 | 0.007127277 |
| M558 | 0.007128074 |
| M600 | 0.008791011 |
| M474 | 0.009609277 |
| M305 | 0.010263691 |
| M555 | 0.015984031 |
| M553 | 0.016536388 |
| M333 | 0.019607355 |
| M517 | 0.02295129 |
| M539 | 0.026132402 |
| M608 | 0.027245944 |
| M422 | 0.029004883 |
| M607 | 0.030515912 |
| M611 | 0.03059874 |
| M576 | 0.035218119 |
| M588 | 0.036833877 |
| M523 | 0.039548612 |
| M472 | 0.04082364 |
| M557 | 0.042934351 |
| M601 | 0.043137615 |
| M546 | 0.044140985 |
| M254 | 0.049131883 |
